# When do plant hydraulics matter in terrestrial biosphere modelling?

**DOI:** 10.1111/gcb.17022

**Published:** 2023-11-14

**Authors:** Athanasios Paschalis, Martin G. De Kauwe, Manon Sabot, Simone Fatichi

**Affiliations:** ^1^ Department of Civil and Environmental Engineering Imperial College London London UK; ^2^ School of Biological Sciences, University of Bristol Bristol UK; ^3^ ARC Centre of Excellence for Climate Extremes and Climate Change Research Centre University of New South Wales Sydney New South Wales Australia; ^4^ Department of Civil and Environmental Engineering National University of Singapore Singapore Singapore

**Keywords:** ecosystem recovery, ecosystem responses, plant hydraulics, terrestrial biosphere model, water stress

## Abstract

The ascent of water from the soil to the leaves of vascular plants, described by the study of plant hydraulics, regulates ecosystem responses to environmental forcing and recovery from stress periods. Several approaches to model plant hydraulics have been proposed. In this study, we introduce four different versions of plant hydraulics representations in the terrestrial biosphere model T&C to understand the significance of plant hydraulics to ecosystem functioning. We tested representations of plant hydraulics, investigating plant water capacitance, and long‐term xylem damages following drought. The four models we tested were a combination of representations including or neglecting capacitance and including or neglecting xylem damage legacies. Using the models at six case studies spanning semiarid to tropical ecosystems, we quantify how plant xylem flow, plant water storage and long‐term xylem damage can modulate overall water and carbon dynamics across multiple time scales. We show that as drought develops, models with plant hydraulics predict a slower onset of plant water stress, and a diurnal variability of water and carbon fluxes closer to observations. Plant water storage was found to be particularly important for the diurnal dynamics of water and carbon fluxes, with models that include plant water capacitance yielding better results. Models including permanent damage to conducting plant tissues show an additional significant drought legacy effect, limiting plant productivity during the recovery phase following major droughts. However, when considering ecosystem responses to the observed climate variability, plant hydraulic modules alone cannot significantly improve the overall model performance, even though they reproduce more realistic water and carbon dynamics. This opens new avenues for model development, explicitly linking plant hydraulics with additional ecosystem processes, such as plant phenology and improved carbon allocation algorithms.

## Graphical abstract

We tested within the terrestrial biosphere model T&C four plant hydraulics modules. We found that plant hydraulics are essential to capture ecosystem dynamics under conditions of plant water stress. However, plant hydraulics by themselves cannot improve the overall model performance in long‐term simulations.

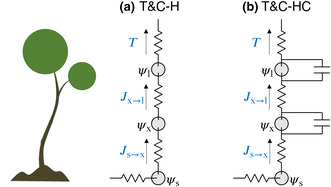

## 1 INTRODUCTION

Terrestrial vegetation currently sequesters about one third of anthropogenic CO_2_ emissions (Friedlingstein et al., 2022), offering an important contribution to achieving carbon reduction targets, and tackling climate change (Griscom et al., 2017). However, as climate changes the capacity of vegetation to offer this critical benefit can decline, because of a projected intensification of climate‐induced plant water stress (Anderegg et al., 2020; Choat et al., 2018; Ukkola et al., 2020). Specifically, the response of vascular plants to environmental change is highly dependent on the integrity and efficiency of their water transport system (Venturas et al., 2017). Water moves from the soil to the atmosphere following a declining water potential gradient according to the cohesion‐tension theory (Tyree, 2003). Plant water transport depends on four main aspects: the strength of the water potential gradient, the conductivity of plant tissues, the internal plant water stores and the opening of plant stomata that regulate water flux from the plants to the atmosphere (Fatichi, Leuzinger, et al., 2016; Fatichi, Pappas, et al., 2016; Mencuccini et al., 2019).

The total potential gradient is determined by the soil and atmospheric water potentials, which in turn depend on soil and atmospheric drought (Porporato & Yin, 2022). Vascular plants have evolved a sophisticated and efficient water transport system (Pittermann et al., 2011), exploiting the available soil water for plant functions (photosynthesis, growth, etc.) while simultaneously regulating water losses due to strong atmospheric water demand (e.g. Anderegg et al., 2016; Oliveira et al., 2021). During intense periods of water stress, plant conducting tissues can lose their efficiency (i.e. conductivity), due to air embolism (Tyree & Sperry, 1989). The almost complete loss of conductivity, commonly termed hydraulic failure, has been identified as a major pathway of plant mortality (e.g. Barigah et al., 2013; Rowland et al., 2015; Urli et al., 2013). Loss of conductivity can also harm the carbon balance of a plant as loss of vascular transport conductivity and plant dehydration reduce carbon sequestration and lead to depletion of the plant carbon reserves (e.g. Choat et al., 2018; McDowell, 2011; Sapes & Sala, 2021). Each plant tissue loses conductivity at different rates, usually in a coordinated way following the paradigm of hydraulic segmentation, with short‐lived plant tissues (i.e. leaves and fine roots) failing earlier than long‐lived plant components (e.g. Charrier et al., 2016; Pivovaroff et al., 2014; Wason et al., 2018).

Leaf stomata serve as the valves regulating water transport (e.g. Körner, 2019). Plant stomata largely close following the hormonal signal of abscisic acid (ABA; e.g. Hsu et al., 2021; Pantin et al., 2013) that can be generated partially in the roots and transported to the leaves, following the transpiration stream, and mostly in leaves (e.g. Buckley, 2019; Hetherington & Woodward, 2003; Zhang et al., 2018) as a response to leaf water potential. When stomata close, the potential gradient from the soil to the leaves drops, leading to reduced transpiration and photosynthesis (e.g. Körner, 2019). This stomatal closure can reduce the damage caused by embolism, which to a large degree is irreversible (Charrier et al., 2016; Sperry, 2013; Venturas et al., 2017). Stomata responses have been hypothesized to operate in a manner that optimizes the plant carbon gains for a given loss of water (e.g. Cowan, 1977; Katul et al., 2010; Manzoni et al., 2011; Medlyn et al., 2011). More recent stomatal optimization work has linked the ‘cost’ of a selective pressure to avoid long‐term damages to the conducting tissues (e.g. Eller et al., 2018; Sperry et al., 2017; Wolf et al., 2016) and maximizing the efficiency of restoring the internal plant water stores (e.g. Peters et al., 2023).

Plants can also buffer demand for water and avoid strong negative potentials by using water from internal water stores such as bulliform cells, water‐storage parenchyma and vascular bundle sheaths (e.g. Luo et al., 2021), water that typically refills during the night and is used the following day (e.g. Huang et al., 2017; Peters et al., 2023). The efficiency of a plant to refill its water stores depends on its water capacitance, as well as its conductance and environmental conditions at night (e.g. Huang et al., 2017). Stomata responses, the hydraulic behaviour of leaves, roots and xylem as well as plant water storage are coordinated, at least to a certain extent, with each other forming a complex plant hydraulic trait spectrum (e.g. Chave et al., 2009; Daz et al., 2016; Manzoni, 2014; Reich, 2014).

Overall, to be able to quantify the responses of water and carbon fluxes, especially as both soil and atmospheric drought intensity and duration are expected to intensify with climate change in various regions (e.g. Ukkola et al., 2020; Vicente‐Serrano et al., 2020), we need to be able to fully capture the entire water pathway from the roots to the atmosphere. To mechanistically quantify these ecosystem water and carbon fluxes, we need to accurately represent: (a) how environmental forcing impacts stomata conductance, (b) how each plant tissue loses (gains) conductivity with declining (increasing) potentials in the short and long term, (c) and how plant water storage depletes and refills, buffering plant water supply and demand.

The recognition of the importance of plant hydraulics has led to the development of models of varying degrees of complexity over the last two decades. Those models open new avenues for a better understanding of the coupled water and carbon cycles. Models span from detailed representations of single plants (e.g. Bohrer et al., 2005; Ruffault et al., 2022), where each plant tissue is modelled explicitly to ecosystem‐scale models (e.g. De Kauwe et al., 2020; Kennedy et al., 2019; Yao et al., 2022), where plant hydraulics are commonly formulated using simplifying conceptualizations. Regardless of the model structure, all models simulate water flow in the xylem based on the cohesion‐tension theory (Tyree, 2003), that is water is moving passively across a gradient of negative potentials that declines from the soil to the atmosphere. Most models simulate the effect of drought on tissue loss of conductivity based on parametric ‘vulnerability curves’ that link water potential to the percentage of conductivity loss of the examined plant tissue (e.g. Venturas et al., 2019).

Among all model formulations proposed, two approaches are the most widely used; one approach neglects xylem and leaf water storage (e.g. Sloan et al., 2021), and one explicitly considers plant storage when solving water flow in plants (e.g. Hartzell et al., 2017; Huang et al., 2017; Xu et al., 2016). A common modelling assumption for both approaches is the reversibility of the vulnerability curves, that is, that reduction of xylem and/or leaf water potential leads to immediate restoration of hydraulic conductance. While this assumption has been criticized, as it implicitly assumes immediate xylem refilling, it is used in most existing plant hydraulics models with few exceptions (e.g. Lu et al., 2022; Mackay et al., 2015).

Models that include plant hydraulics capture well the seasonal and diurnal variability of water and carbon fluxes, in temperate (e.g. Mirfenderesgi et al., 2016), boreal (e.g. Lambert et al., 2022) and tropical (e.g. Yao et al., 2022) ecosystems. When plant hydraulics are added to terrestrial biosphere models, they alter their sensitivity to hydrological and atmospheric droughts (e.g. Liu et al., 2020), as well as the diurnal dynamics of water and carbon fluxes (e.g. Hartzell et al., 2017). Linking plant hydraulics modules to phenological changes and the risk of mortality due to hydraulic failure has also been found to improve model performance (e.g. Xu et al., 2016).

Given the potential role of treating plant hydraulics in carbon and water fluxes explicitly and that no commonly agreed model formulation exists, it is important to evaluate the role of different levels of complexity in representing plant hydraulics and their intrinsic importance in different biomes and climatic conditions. This calls for a systematic evaluation of plant hydraulics modelling approaches, which frames the scope of this aricle.

To achieve this research scope, we expand the capabilities of the terrestrial biosphere model T&C by introducing four different plant hydraulics variants with an increasing degree of complexity. Differently from other studies (e.g. Kennedy et al., 2019; Xu et al., 2016), the experiment exclusively changes plant hydraulic representation in the same model, to assess their importance in determining ecosystem‐scale water and carbon dynamics. The specific research questions addressed here are as follows:

1. How do different plant hydraulics representations alter plant responses to water stress?
2. What is the effect of plant water storage and xylem damage in the water and carbon fluxes across time scales?
3. Can the variability of whole ecosystem‐scale water and carbon dynamics be explained by plant hydraulics or not? and what is plant hydraulics potential for improving ecosystem models?

## 2 METHODS

### 2.1 Model description

In this study, we introduce four plant hydraulic modules to the terrestrial biosphere model T&C (Fatichi et al., 2012). T&C is a mechanistic terrestrial biosphere model that couples a land surface energy balance scheme with a hydrological, vegetation and soil biogeochemical module (Fatichi et al., 2019). T&C has been used globally to simulate water and carbon dynamics and their sensitivity to environmental forcing for many ecosystem types (e.g. Fatichi et al., 2021; Fatichi, Leuzinger, et al., 2016; Fatichi, Pappas, et al., 2016; Moustakis et al., 2022; Paschalis et al., 2018, 2022), including extreme environments (Fugger et al., 2022; Fyffe et al., 2021; Mastrotheodoros et al., 2020) and urban ecosystems (e.g. Meili et al., 2021; Zhang et al., 2021, 2022, 2023). The model follows a physics‐based formulation that closes the coupled water, carbon, energy and nutrient (N/P/K) balances. Model details can be found in Fatichi et al. (2012, 2019) and Meili et al. (2020).

Importantly, T&C conceptualizes the canopy structure using a two‐big leaf scheme, where the canopy is split into a sun and a shaded leaf. Leaf photosynthesis is simulated with an adaptation of the widely used models of Farquhar (1989) for the C3. T&C can also simulate the C4 photosynthetic pathway (not used in this study) following Collatz et al. (1992) and Bonan et al. (2011). Stomatal conductance is modelled following the Leuning model (Leuning, 1990), which was previously found to provide similar results to the other widely used empirical or optimality‐based conductance models (Paschalis et al., 2017). In fact, to confirm that the choice of Leuning model will not affect our results, we also implemented within the T&C model the optimality model of Medlyn et al. (2011). The differences for all sites investigated here were negligible with absolute average differences in hourly simulated plant transpiration of 0.005 mm h^−1^ and hourly gross photosynthesis of 0.1 μmol m^−2^ s^−1^ and thus not further discussed.

Simulations with T&C can have multiple vegetation types that cover a preassigned fraction of the land. Vegetation cover is constant throughout the simulation, and different types do not compete for area. Different vegetation types compete for soil water, but their cover fraction is static. Each vegetation type is conceptualized by six carbon pools (leaves, living sapwood, fine roots, carbohydrate reserves, fruits and flowers and heartwood). Throughout the manuscript whenever referring to dynamic vegetation, we mean dynamic evolution of the carbon pools in time, and not competition between vegetation types for space. The carbon pools are initialized with a 30‐year long spin‐up simulation using either observed meteorological forcing if available, or a random permutation of observed meteorological years when observations are less than 30 years long. Throughout the manuscript, we also avoid the term plant functional types, as all plant traits in T&C can be set by the modeller.

The necessary meteorological forcing for the T&C model is hourly values of downwelling short‐ and long‐wave radiation split into direct and diffuse, atmospheric CO_2_ concentration, wind speed, temperature and relative humidity. Temperature and relative humidity are used to calculate the vapour pressure deficit (VPD). Shortwave radiation is further split into wavelength bands to separate the photosynthetic active radiation component used by vegetation. Radiation is split into direct and diffuse, as well as in different wavebands using the procedures used in the AWEGEN weather generator (Fatichi et al., 2011; Peleg et al., 2017).

### 2.2 Representing drought stress within T&C

In the default T&C version without plant hydraulics, plant water stress is modelled with a multiplicative reduction factor $f_{s}$, applied on the potential (unstressed) leaf carbon assimilation rate. The reduction factor $f_{s}$ is modelled using a sigmoid function. Specifically:

(1) $$f_{s}=\exp\left(-q{\left|\psi_{s}\right|}^{p}\right)$$

where $\psi_{s}=P\left(\sum_{i}w_{i}\theta \left(i\right)\right)$ is a root biomass weighted average soil water potential. $\theta \left(i\right)$ the soil water content of the *i*‐th soil layer, $w_{i}$ the fraction of fine roots in the *i*‐th layer, $P\left(\theta \right)$ the water retention curve and *p*, *q* the two empirical parameters that define the shape of the sigmoid curve. This reduction factor directly affects both the carbon assimilation rate and the stomatal conductance which follows Leuning's semiempirical formulation:

(2) $$g_{s}=g_{0}+a_{1}\frac{f_{s}A_{n}^{\mathrm{pot}}}{\left(c_{i}-\Gamma \right)}\frac{1}{\left(1+D/D_{0}\right)}$$

where $a_{1}$ [‐] a model parameter describing the sensitivity of stomatal conductance to leaf photosynthesis, $g_{0}$ [mol CO_2_ m^2^ s^−1^] a residual stomatal conductance as net assimilation rate reaches zero, $A_{n}^{\mathrm{pot}}$ [mol CO_2_ m^2^ s^−1^] the potential (unstressed) plant photosynthesis, $c_{i}$ [molar fraction] the leaf internal (intercellular spaces) CO_2_ concentration, Γ the CO_2_ compensation point, D [Pa] the VPD and $D_{0}$ [Pa] a parameter determining the sensitivity of stomatal conductance to VPD (*D*). The net assimilation under water stress is $A_{n}^{\text{stressed}}=f_{s}A_{n}^{\mathrm{pot}}$. Stomatal conductance is thus limited by both soil water limitation and atmospheric drought, as well as all meteorological forcing affecting $A_{n}^{\mathrm{pot}}$, such as light intensity and temperature.

### 2.3 Introducing plant hydraulics within T&C

**FIGURE 1:**
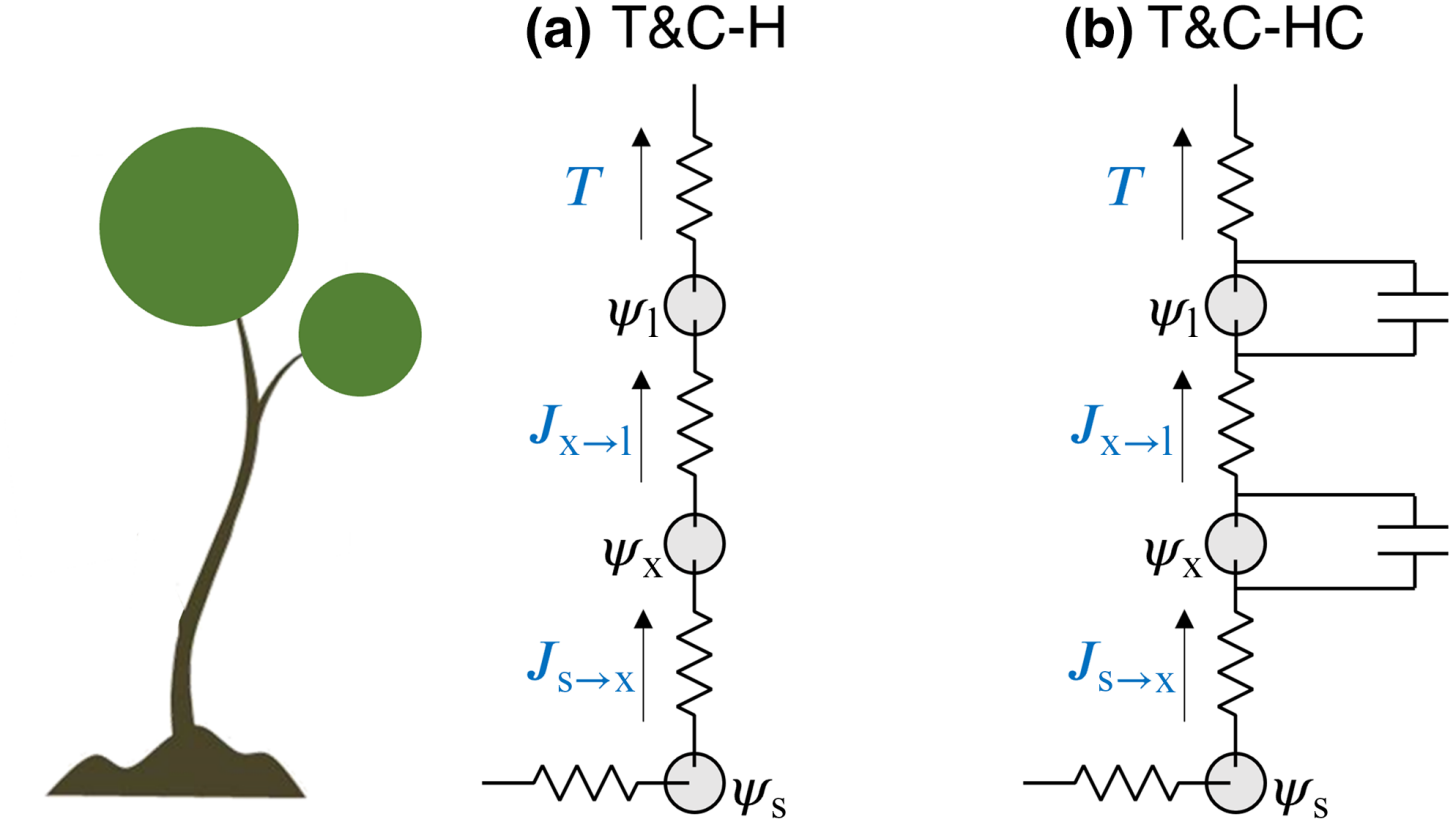
Schematic representation of plant hydraulics models. (a) Model without capacitance T&C‐H and (b) model including capacitance T&C‐HC.

All variants of plant hydraulics share the same simplifications, conceptualizing the state of a plant with a single computational node for the trunk and stem xylem, and a single node for all leaves, (Figure 1). The two plant hydraulics model variants we introduce are as follows: T&C‐H, which neglects xylem and leaf capacitance, and T&C‐HC, which includes the capacitance terms.

In both T&C‐H and T&C‐HC, nodes are described by their hydraulic conductance ($k_{x}$, $k_{l}$, for xylem and leaves accordingly) and their corresponding water potential ($\psi_{x}$, $\psi_{l}$ in [MPa]). For this study, we neglect the role of gravitational potential for parsimony as results when including it were not significantly different (not shown here). In T&C‐HC, xylem and leaves are also described by their water content ($V_{x}$, $V_{l}$ in units of water volume per unit land area).

### 2.4 Plant hydraulics without capacitance: T&C‐H

In T&C‐H, we neglect xylem and leaf water capacitance and compute $\psi_{x}$ and $\psi_{l}$ at an hourly time scale, conditional to a known soil water potential $\psi_{s}$ by solving the following system of equations numerically:

(3) $$J_{s\to x}=\bar{k_{s\to x}}\left(\psi_{s}-\psi_{x}\right)=T\left(\psi_{s},\psi_{l},M\right)$$

(4) $$J_{x\to l}=\bar{k_{x\to l}}\left(\psi_{x}-\psi_{l}\right)=T\left(\psi_{s},\psi_{l},M\right)$$

where $J_{s\to x}$, $J_{x\to l}$ [m s^−1^] are the water fluxes from the soil to the xylem node and the xylem node to the leaf accordingly, $\bar{k_{s\to x}}$ [m s^−1^ MPa^−1^] is the geometric mean between the soil‐to‐root and xylem conductance, and $\bar{k_{x\to l}}$ [m s^−1^ MPa^−1^] is the geometric mean between the xylem and leaf conductance. M expresses the meteorological forcing (temperature, wind speed, radiation, atmospheric pressure and VPD), and T [m s^−1^] is the transpiration rate that is conditional to both soil and leaf water potential due to its proportionality with stomatal conductance, that is $T\propto g_{s}D$.

The xylem conductance $k_{x}$ is parameterized as a function of the xylem water potential:

(5) $$k_{x}=\exp\left(-q_{x}{\left|\psi_{x}\right|}^{p_{x}}\right)k_{x}^{\max}$$

where the $k_{x}^{\max}$ [m s^−1^ MPa^−1^] parameter is the maximum xylem conductance when $\psi_{x}=0$. Scaling of the tissue level xylem conductivity $k_{x}$ in [kg m^−1^ s^−1^ MPa^−1^] to $k_{x}$ is done as $k_{x}=k_{x}{\mathrm{hA}}_{x}^{-1}\rho^{-1}$, where h [m], the plant height, $A_{x}$ [m^2^] the average xylem cross‐sectional area per unit ground area and ρ [kg m^−3^] the water density. $q_{x}$ and $p_{x}$ are two empirical coefficients describing the shape of the xylem vulnerability curve.

Similarly, the leaf conductance is modelled as a function of the leaf water potential as:

(6) $$k_{l}=\exp\left(-q_{l}{\left|\psi_{l}\right|}^{p_{l}}\right)k_{l}^{\max}$$

where the $k_{l}^{\max}$ [m s^−1^ MPa^−1^] parameter is the maximum leaf conductance and $q_{l}$ and $p_{l}$ are the two empirical coefficients describing the shape of the leaf vulnerability curve. The soil‐to‐root hydraulic conductivity $K_{r}$ [m s^−1^ MPa^−1^] was parameterized as a function of the soil water potential, its hydraulic conductivity and the root properties as in Hölttä et al. (2009):

(7) $$k_{r}=c_{f}k_{s}\left(\psi_{s}\right)\frac{2\pi R_{l}}{\log\left(r_{c}/r_{r}\right)}$$

where $k_{s}\left(\psi_{s}\right)$ [m s^−1^] the (unsaturated) soil hydraulic conductivity at $\psi_{s}$, $R_{l}$ [m root m^−2^ ground] the root length index, $r_{c}$ [m] the radius of the cylinder of soil to which the root has access to, $r_{r}$ [m] the root radius and $c_{f}=1/\mathrm{\rho g}$ a dimension conversion factor.

To close the system of equations, a model for stomatal conductance is needed to compute plant transpiration. We opt for a model similar to Tuzet et al. (2003) that links stomatal conductance to photosynthetic rate, soil and leaf water potential. Specifically, we opt for a formulation where we include both ‘nonstomatal’ and stomatal limitations (e.g. Drake et al., 2017; Zhou et al., 2014) regulating stomatal conductance, as they have been found to be essential for capturing short‐ and long‐term responses to water limitation. The stomatal conductance is defined as:

(8) $$g_{s}=g_{0}+a_{1}\frac{A_{n}^{\mathrm{pot}}f_{s}}{c_{i}-\Gamma }f_{l}$$

where $f_{l}$, $f_{s}$ are two reduction factors. $f_{s}$ is the ‘nonstomatal’ limitation that reduces net assimilation identically to the T&C model and depends on soil water potential alone, that is $A_{n}^{\text{stressed}}=A_{n}^{\mathrm{pot}}f_{s}$. $f_{l}$ is a stomatal conductance reduction factor that depends on the difference between soil water potential and leaf water potential, triggered by atmospheric water demand. Conceptually $f_{l}$ provides similar functionality to the term ${\left(1+D/D_{0}\right)}^{-1}$ in the Leuning model, but it depends on the dynamics of the plant hydraulic system. Mechanistically, the reduction factors are conceptualizations of the root and leaf ABA production that leads to stomatal closure during water stress (Tardieu & Davies, 1993). The formulation of $f_{l}$ is an exponential function:

(9) $$f_{l}=\exp\left(-a_{l}{\left(\left|\psi_{s}-\psi_{l}\right|\right)}^{\gamma_{l}}\right)$$

where $a_{l}$, $\gamma_{l}$ empirical parameters. The simplest stomatal conductance model possible that expresses conductance solely on leaf water potential, that is independent of soil water potential, carbon assimilation and CO_2_ concentration, was tested and led to unrealistically high conductance values at night, as it could not capture the stomatal responses to light. Two alternative stomatal conductance expressions were also tested that decoupled $g_{s}$ from ${\mathrm{psi}}_{s}$, removing the ‘nonstomatal’ limitation $\left(g_{s}=g_{0}+a_{1}^{*}\left(A_{n}^{\mathrm{pot}}/c_{i}-\Gamma \right)f_{l}^{*},\text{with}\;f_{l}^{*}=\exp\left(-a_{s}{\left(\left|\psi_{l}\right|\right)}^{\gamma_{s}}\right)\right)$, and decoupled it from both $\psi_{s}$ and $c_{i}$ ($g_{s}=g_{0}+a_{1}^{*}A_{n}^{\mathrm{pot}}f_{l}^{*}$). The results were very similar to the results we report in the manuscript, and thus, we do not further discuss them, even though they are all good alternatives. For completeness, we provide a comparison of the various stomatal conductance models in the Supporting Information (Figure S1).

For most cases, the system of Equations (3) and (4) was solved numerically using the computationally efficient Powell's dog leg method (Powell, 1970). During periods of drought stress, the method occasionally gave erroneous results. When the dog leg method had an accuracy less than 1%, then xylem and leaf water potentials were estimated by minimizing the problem ${\text{argmin}}_{\psi_{x},\psi_{l}}=|\bar{k_{s\to x}}\left(\psi_{s}-\psi_{x}\right)-T\left(\psi_{s},\psi_{l},M\right)|+|\bar{k_{x\to l}}\left(\psi_{x}-\psi_{l}\right)-T\left(\psi_{s},\psi_{l},M\right)|$ conditional to $\psi_{s}\geq \psi_{x}\geq \psi_{l}$ using the more robust but computationally more expensive interior point algorithm. The high computational cost was because the minimization was performed as a global optimization problem with 100 randomly generated initial values for the interior point algorithm to ensure convergence.

### 2.5 Plant hydraulics with plant water storage: T&C‐HC

Neglecting plant water capacitance, as in T&C‐H, might lead to the wrong estimation of water and carbon dynamics at short time scales (i.e. subdaily) when demand for water can be buffered using internal plant water storage. As a result, we introduce the second variant, which explicitly considers water storage in the xylem and leaves, and solve their water potential similar to Xu et al. (2016). Specifically, to compute plant water flow we solve the coupled system of ordinary differential equations

(10) $$\frac{{\mathrm{dV}}_{x}}{\mathrm{dt}}=J_{s\to x}-J_{x\to l}$$

(11) $$\frac{{\mathrm{dV}}_{l}}{\mathrm{dt}}=J_{x\to l}-T$$

where $V_{x}$ and $V_{l}$ are the xylem and leaf water content, respectively in [m], expressed as volume of water per unit land area. $V_{x}$ and $V_{l}$ relate to $\psi_{x}$ and $\psi_{l}$ with a simple linear pressure–volume curve for parsimony

(12) $$\psi_{x}=c_{x}\left(1-\frac{V_{x}}{V_{x}^{\max}}\right)$$

(13) $$\psi_{l}=c_{l}\left(1-\frac{V_{l}}{V_{l}^{\max}}\right)$$

where $V_{x}^{\max}$ is the maximum water content in the xylem and $V_{l}^{\max}$ is the equivalent for the leaves. $V_{x}^{\max}$ expressed in units of water volume per unit ground area is ${\mathrm{hA}}_{x}n_{x}$, where h [m] is the plant height, $A_{x}$ [m^2^ m^−2^] the average cross‐sectional xylem area per unit ground area and $n_{x}$ [‐] the water holding capacity of the xylem conduits plus nonconducting tissues. Similarly, $V_{l}^{\max}$ is defined as $V_{l}^{\max}=\mathrm{LAI}\times \mathrm{LMA}\frac{1-\text{LDMC}}{\text{LDMC}}\frac{1}{\rho }$, where LAI [m^2^ m^−2^] is the leaf area index, LMA [kg m^−2^] is the leaf mass per area, and LDMC [g g^−1^] is the ratio of dry to fresh leaf density. $c_{x}$ and $c_{l}$ are the minimum xylem and leaf water potentials when the $V_{x}$ and $V_{l}$ reach 0. The system of equations was integrated numerically with a time‐adaptive stiff ode solver (Runge–Kutta–Fehlberg method 2–3 s). The lower boundary condition of the soil water potential was coupled asynchronously with the soil water solver and the energy balance solution of T&C at a time step of 1 min. More realistic but less parsimonious functions for the pressure–volume curves (Tyree & Hammel, 1972) were also tested, resulting in minor differences, and thus not further analysed in the rest of the article.

### 2.6 Plant hydraulics with xylem damage: T&C‐H‐d, T&C‐HC‐d

Finally, we introduced hydraulics legacies to the damage of the water‐conducting tissues, assuming no refilling and thus no immediate conductivity restoration after soil moisture and soil water potential return to noncritical levels. Specifically, the conductivity for the xylem was computed as:

(14) $$k_{x}=\int_{0}^{\infty }k_{x}^{\alpha }\left(\psi_{x}^{\min}\left(\alpha \right)\right){\mathrm{dp}}_{\alpha }$$

where $k_{x}^{\alpha }\left(\psi_{x}^{\min}\left(\alpha \right)\right)$ is the minimum xylem conductivity during its life span α, when the minimum water potential occurred, and $p_{\alpha }$ the probability distribution of the xylem age, that is the distribution of the time since living xylem tissues were first constructed. Simply put, in this formulation xylem tissue cannot restore its conductivity unless new xylem tissues are built. The exact same formulation is also used for $k_{l}$.

As T&C resolves vegetation dynamically, and it has two separate carbon pools for leaves and xylem (living sapwood), $p_{\alpha }$ can be dynamically computed as:

(15) $$\frac{\partial }{\partial t}p_{\alpha }\left(t\right)=-\frac{\partial }{\partial \alpha }p_{\alpha }\left(t\right)-\mu_{x}\left(\alpha ,t\right)p_{\alpha }\left(t\right)$$

with a boundary condition

(16) $$p_{0}\left(t\right)=\lambda_{x}$$

where $\mu_{x}\left(\alpha ,t\right)$ is the turnover rate of the tissue for an age class of age α and $\lambda_{x}$ is the new tissue being built. In the model, we assumed that turnover was applied to the oldest tissues first. New xylem and leaves being built have their maximum possible capacity (i.e. there are born full of water). The partial differential equation was solved numerically, and both $\mu_{x}\left(\alpha ,t\right)$ and $\lambda_{x}$ were computed by the dynamic vegetation component of T&C. Simulations with hydraulic failure legacy were performed for both T&C‐H and T&C‐HC (hereafter T&C‐H‐d and T&C‐HC‐d).

### 2.7 Study sites

We analysed six sites with woody vegetation, spanning a wide range of climates. The choice of the sites was dictated by maximizing biome representativeness and was limited by data availability, and particularly data necessary to parameterize the plant hydraulic models we implemented. All six sites were equipped with eddy covariance systems monitoring half‐hourly water and carbon fluxes, and five sites had also sapflow sensors reporting sapflux at half‐hourly steps. All data were rescaled to hourly for model validation. The sites from wettest to driest are Br‐CAX (tropical evergreen rainforest, Brazil), GF‐Guy (tropical evergreen rainforest, French Guyana), US‐UMB (temperate deciduous forest, USA‐MI), FI‐Hyy (evergreen boreal forest, Finland), FR‐Pue (evergreen conifer Mediterranean forest, France) and US‐SRM (semiarid shrubland, USA‐AZ). All sites except Br‐CAX are part of FLUXNET. Details for all sites can be found in Table 1. All sites except US‐SRM were conceptualized with a single vegetation type in T&C covering the entire area. For US‐SRM, where deciduous and evergreen shrubs co‐exist and are sparse, we used two vegetation types: one deciduous covering 35% of the land area and one evergreen covering 20%. The rest 50% was considered bare soil.

**TABLE 1:** Site description. Climate averages correspond to the data period used for the simulations only.

	BR‐CAX	GF‐Guy	US‐UMB	FI‐Hyy	FR‐Pue	US‐SRM
Longitude [degrees]	−51.45	−52.91	−84.71	24.29	3.59	−110.86
Latitude [degrees]	−1.75	5.28	45.55	61.84	43.74	31.82
Ecosystem	Tropical evergreen forest	Tropical evergreen forest	Deciduous Broadleaf forest	Evergreen needleleaf forest	Evergreen Broadleaf forest	Woody Savanna
Soil texture	Sandy loam	Clay loam (hills); sandy clay loam (plateau)	Sand	Loamy sand	Clay loam	Mixed sandy loam and loamy sand
Temperature [°C]	25.9	25.6	7.2	4.4	13.7	19
Precipitation [mm year^−1^]	2086	2920	613	615	929	1712
Vapour pressure deficit [Pa]	428	571	397	270	659	328
Simulation years	1999–2003 and 2014–2016	2004–2016	2000–2014	1999–2014	2000–2014	2004–2014
Eddy covariance years	1999–2003	2004–2014	2000–2014	1999–2014	2000–2014	2004–2014
Sap flow years	2014–2016	2014–2016	2010–2016	2013–2016	2000–2015	[‐]
Tree species with sap flow sensors	*Manilkara bidentata*	*Sloanea* sp.	*Acer rubrum*	*Pinus sylvestris*	*Quercus ilex*	
	*Swartzia racemosa*	*Vacapoua americana*	*Betula papyrifera*	*Betula pendula*		
	*Eschweilera coriacea*	*Licania membranacea*	*Populus grandidentata*			
	*Licania membranacea*	*Oxandra asbeckii*	*Pinus strobus*			
	*Eschweilera grandiflora*	*Iryanthera sagotiana*	*Quercus rubra*			
	*Protium tenuifolium*	*Goupia glabra*				
	*Licania octandra*					
	*Pouteria anomala*					
Site/data doi	https://daac.ornl.gov/LBA/guides/CD32_Fluxes_Brazil.html	10.18140/FLX/1440165	10.18140/FLX/1440093	10.18140/FLX/1440158	10.18140/FLX/1440164	10.18140/FLX/1440090

Hourly meteorological (radiation, wind speed, temperature, relative humidity and air pressure) was obtained by the continuous quality controlled data produced for the PLUMBER2 model intercomparison project (Ukkola et al., 2022), for all sites except Br‐CAX. Forcing for Br‐CAX was derived from both a local weather station (1999–2003) at the flux‐tower site (Restrepo‐Coupe et al., 2021) and the data provided by SAPFLUXNET for 2014–2018 (Poyatos et al., 2021). Water and carbon flux data were also obtained by the repositories of PLUMBER2 and originated from the FLUXNET2015 dataset (Pastorello et al., 2020). Eddy covariance data for Br‐CAX were from Restrepo‐Coupe et al. (2021). Sapflow data were obtained by SAPFLUFLUXNET (Poyatos et al., 2021). The key model parameters relevant to this article can be found in Table S1. The remaining parameters can be found in the parameter files for the T&C models (see Data Availability Statement for model access options). All parameters for the original T&C model were obtained in previous studies (e.g. Fatichi, Leuzinger, et al., 2016; Fatichi, Pappas, et al., 2016; Moustakis et al., 2022; Paschalis et al., 2022) after extensive model evaluation.

The model parameters describing soil water stress $f_{s}$ (Table S1) were identical for all model variants, including the original T&C, and obtained in previous studies (e.g. Moustakis et al., 2022; Paschalis et al., 2022). The parameters describing stomatal closure due to the drop of water potential from soils to leaves $\left(\psi_{l}-\psi_{s}\right)$ were chosen such that a 1.0 (0.2) MPa drop in this water potential gradient (neglecting gravitational potential differences) leads to a 50% (10%) reduction in stomatal conductance. Parameters for xylem and leaf conductivity, as well as the parameters describing the vulnerability curves, were obtained when possible from observations (see Table S1). Specifically, the parameters were obtained from the publications presented in Table S1, where parameter values were given for the same six sites as in our analysis. Both conductivity values and the shape of the vulnerability curves were obtained by digitization of the figures reported in the references in Table S1. We additionally performed parameter perturbation within a 50% margin via trial and error to ensure good agreement between models and observations, but no further automated model calibration was conducted.

### 2.8 Numerical experiments

For all six sites, we performed three numerical experiments. The first experiment (E1) was a single, synthetic dry‐down experiment. For each site, we extracted the 3 months where vegetation was most active (i.e. monthly observed gross primary productivity [GPP]). We then created an average ‘warm and sunny’ day. In doing so, we assumed a diurnal pattern of meteorological forcing where temperature and radiation were set equal to the 75% percentile of the observed data for each hour for the three most active months. All other weather variables, except relative humidity, were set to the median of the respective hour. Two values for relative humidity were chosen, one set equal to the observed 10% percentile (E1a) and one set to the 90% percentile (E1b), to explore the impact of atmospheric drought. Those values of relative humidity generated a typically high and low, yet realistic, VPD at each site. VPD was computed as a function of the temperature and the relative humidity (i.e. $D=\left(1-\mathrm{RH}\right)e_{\mathrm{sat}}\left(T_{a}\right)$, where RH the relative humidity in [0–1] and $e_{\mathrm{sat}}\left(T_{a}\right)$ the saturated vapour pressure at temperature $T_{a}$). The same daily forcing was repeated for 250 days. For this experiment, the dynamic vegetation was deactivated in T&C and LAI and fine roots were set equal to the average LAI and fine root biomass of the most active 3 months. These average values were computed using the original T&C model. Specifically, the original version of T&C was used to simulate the entire observation data (i.e. long‐term simulations). Afterwards, average LAI was calculated by those long‐term simulations. This experiment is intended to identify model differences during drought intensification without other confounding factors and to provide insights into the different roles of soil and atmospheric drought accordingly. The variables that were analysed include the rate of GPP and transpiration decline following the onset of drought (defined here as the start of the precipitation‐free period) and the diurnal distribution of GPP, plant transpiration and leaf water potentials. Diurnal patterns were analysed under low and high soil drought conditions and low and high atmospheric drought conditions.

The second experiment (E2) was also a dry‐down similar to experiment E1, but this time followed by an intense rainfall event adequate to saturate the soil fully. The duration of the dry period was considered such that the water stress reduction factor for the T&C model reached $f_{s}=0.2$. In E2, dynamic vegetation was enabled; that is, the vegetation carbon pools were not fixed but were let to evolve in time, responding to environmental forcing. E2 is intended to provide further insights into model behaviour in both drought intensification and relief. Similar to E1, both high VPD (E2a) and low VPD (E2b) conditions were considered. The key variables analysed in the E2 experiments include the recovery of GPP, transpiration and LAI following a major dry‐down period.

Finally, experiment 3 (E3) used the observed hourly (rescaled from half‐hourly observations) meteorological time series for the entire record to perform multiyear simulations. E3 is intended to quantify the importance of different plant hydraulic formulations in the overall ecosystem response, as it will be simulated by a terrestrial biosphere model in any environmental change experiment. The variables that were analysed in E3 include the seasonal, diurnal and daily performance of all model formulations regarding GPP and evapotranspiration, as well as the simulation of the surface conductance (i.e. lumped stomatal and soil conductance terms). We also analysed the sensitivity of the models to multiple environmental forcing. Surface conductance ($G_{s}$), for both models and observations, was computed by inverting the Penman–Monteith equation (Monteith, 1965) assuming neutral atmospheric conditions (i.e. $G_{s}=\left(G_{a}\mathrm{\gamma \lambda E}\right)/\left(\Delta \left(R_{n}-G\right)-\left(\Delta +\gamma \right)\mathrm{\lambda E}+G_{a}\rho_{a}c_{p}D\right)$, with $G_{a}$ the neutral aerodynamic conductance, D the VPD, λE the latent heat flux, γ the psychrometric constant, $R_{n}$ the net radiation, G the ground heat flux, $\rho_{a}$ the dry air density, $c_{p}$ the specific heat capacity of air and Δ the rate of change of saturation specific humidity with air temperature). $G_{s}$ is closely related but not identical to stomatal conductance as it expresses the total water vapour conductance from the land surface to the atmosphere, and not only through transpiration. Model sensitivity to environmental forcing was computed based on the out‐of‐bag importance values, which were calculated using a random forest regression algorithm (Loh, 2002). Specifically daily environmental forcing, including temperature, relative humidity, VPD, soil water content, radiation and wind speed, as well as LAI, were used in a random forest regression model to predict the simulated outputs for GPP, plant transpiration and stomatal conductance. The importance of each variable in explaining GPP, transpiration and stomatal conductance was computed using out‐of‐bag importance values. An alternative way to compute the importance using Shapley values (Lundberg & Lee, 2017) is reported in the Supporting Information for completeness. Random forest regression and calculation of out‐of‐bag importance and Shapley values were done using the MATLAB 2023a Statistics and Machine Learning Toolbox.

For all experiments, the initial conditions assumed a fully saturated soil. In E1, we used the models T&C, T&C‐H and T&C‐HC. For E2 T&C‐HC, T&C‐HC‐d and for E3 all model variants.

## 3 RESULTS AND DISCUSSION

We structure the discussion of the results by first focussing on the idealized experiments, to understand the model results fully, without introducing uncertainties linked to weather stochasticity, and then we investigate model applicability at real case studies.

### 3.1 Model responses as drought progresses

**FIGURE 2:**
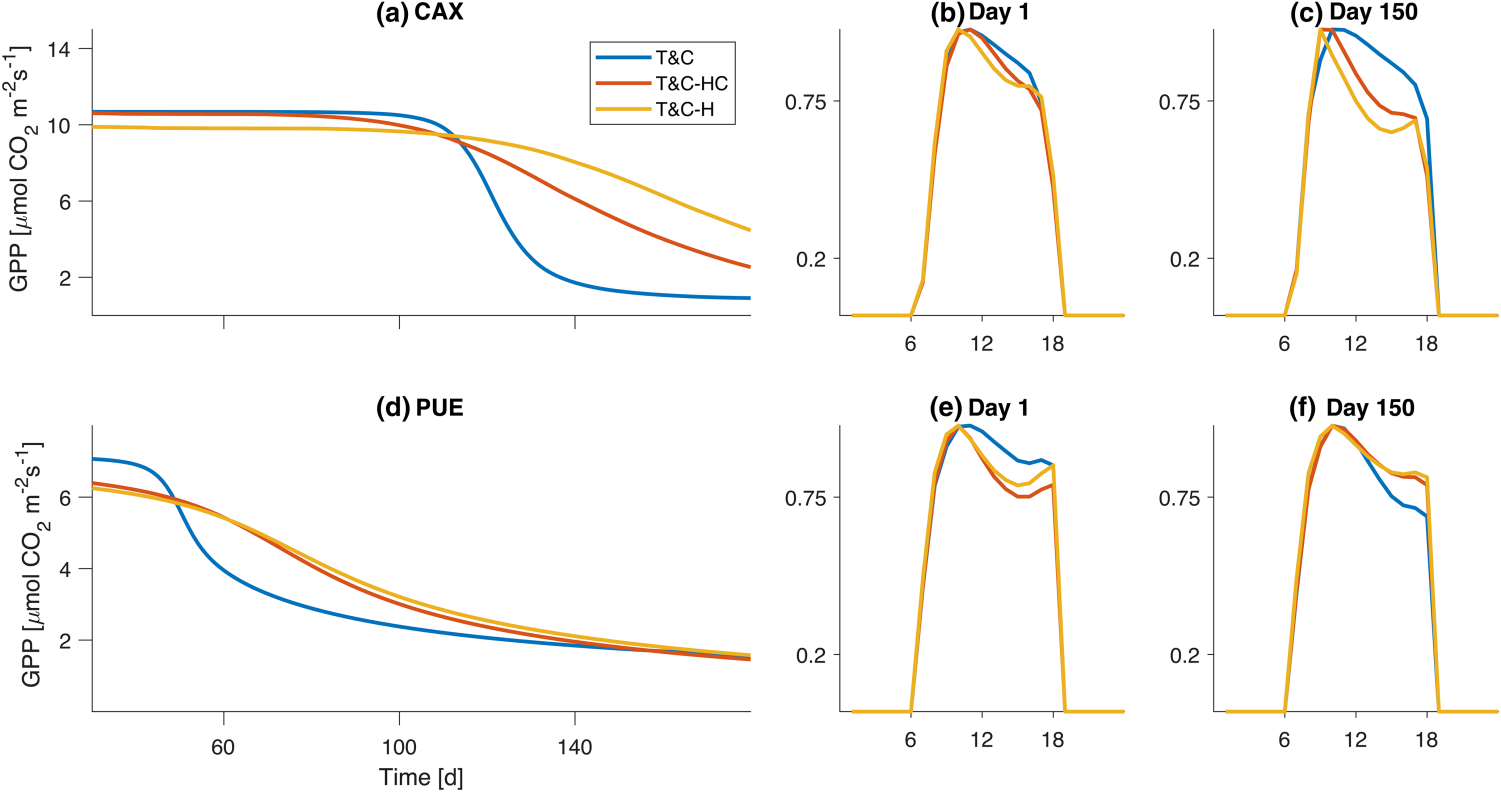
Simulated daily gross primary productivity (GPP) during the dry‐down experiment (E1a) for (a) Br‐CAX and (d) Fr‐PUE. Normalized diurnal average GPP fluxes before drought onset [(b) for Br‐CAX and (e) for Fr‐PUE] and during a fully developed drought [(c) for Br‐CAX and (f) for Fr‐PUE].

The different model variants produce distinct signatures in the responses of both water and carbon fluxes as soils dry (Figure 2). The main patterns are similar for water and carbon fluxes and for all sites. For the sake of clarity here, we will focus on two extreme cases here, a tropical rainforest and a semiarid Mediterranean forest (Figure 2). All other results are presented in the Supporting Information for completeness (Figures S3–S6).

Looking at a synthetic dry‐down event, when plant hydraulics are included in T&C, the decline of photosynthetic rate and evapotranspiration is less abrupt compared with the original model. Comparing the time duration needed for GPP to drop from 90% of its initial rate to 50%, in T&C‐H, it took 43.8 ± 75.3 (mean ± standard deviation across sites) days longer whereas it took 22.8 ± 51.3 in T&C‐HC, in E1a, when VPD is high. Similarly, it took 43.8 ± 68.4 and 22.8 ± 45.2 days longer for T&C‐H and T&C‐HC accordingly when VPD is low (E1b). The reason for this is that the additional xylem and leaf resistances introduced in T&C‐H and T&C‐HC increase as the soil water potential decreases leading to an earlier stomatal closure compared with the original T&C model. In more detail, in T&C, soil water stresses vegetation solely through the $f_{s}$ reduction factor, whereas in T&C‐H and T&C‐HC, there is an additional reduction that comes from the increased resistance to water flow from the soil to the leaves (Figure S2). This additional resistance limits transpiration in T&C‐H and T&C‐HC earlier than in the T&C formulation and can thus deplete the available soil water at a slower rate. The slow rate was mostly independent of atmospheric drought, as the delay simulated in E1a and E1b was almost identical.

**FIGURE 3:**
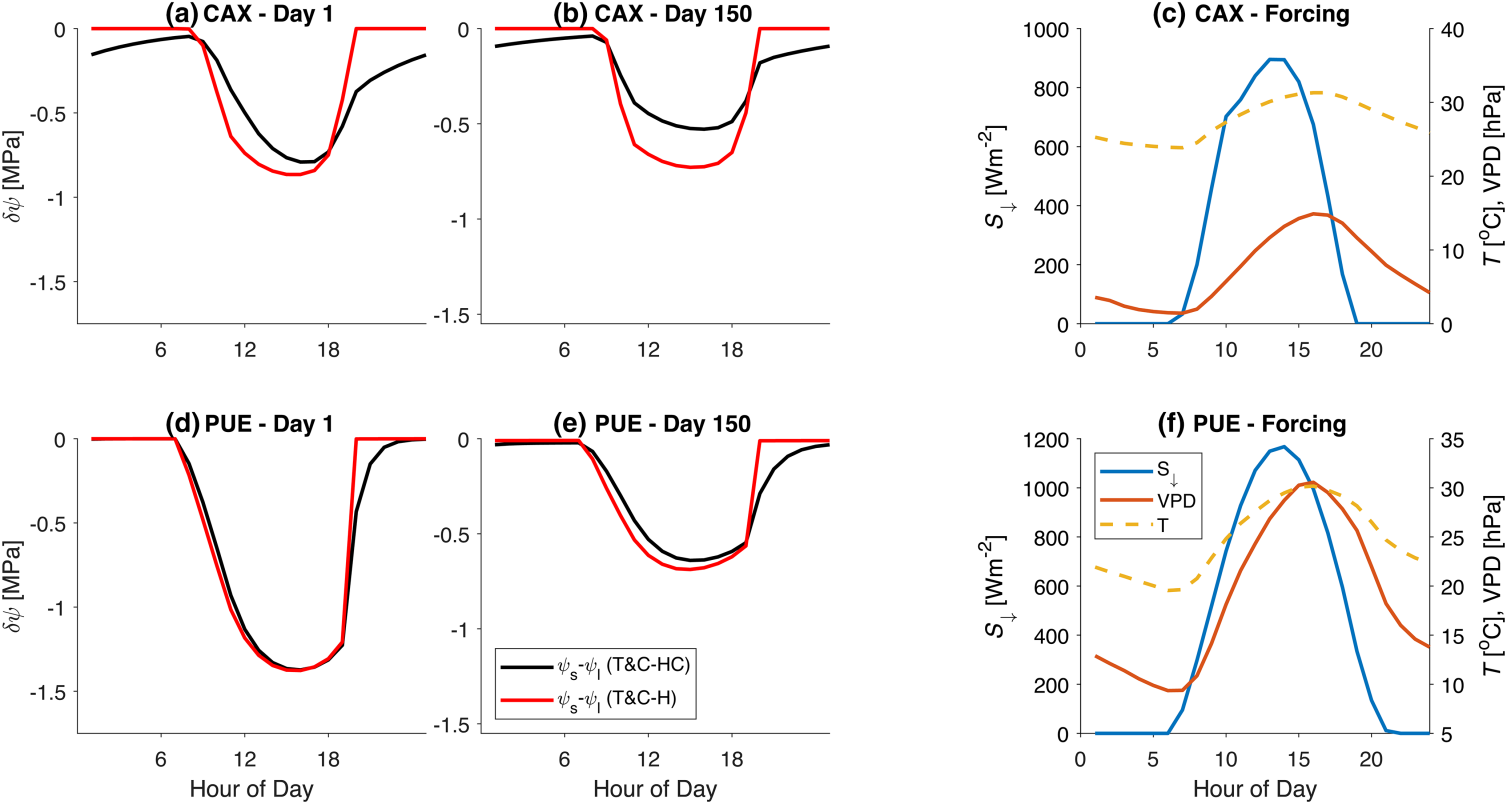
Simulated diurnal difference between soil ($\psi_{s}$) and leaf ($\psi_{l}$) water potential before drought onset [(a) for Br‐CAX and (d) for Fr‐PUE] and during a fully developed drought [(b) for Br‐CAX, (e) for Fr‐PUE]. Simulations correspond to the dry‐down experiment (E1a). Diurnal variability of the meteorological forcing used for the dry‐down experiments [(c) for Br‐CAX, (f) for Fr‐PUE].

Counter‐intuitively, when plant water storage was considered in T&C‐HC, the onset of drought stress was on average faster compared with T&C‐H. The main reason for this behaviour is that when soil moisture is not limiting, transpiration in T&C‐HC is higher than in T&C‐H. That is due to the stored water in leaves and the xylem that can be easily used for transpiration. This happens in the early morning hours when leaf water potential is higher in T&C‐HC than in T&C‐H (Figure 3a,d). This higher water potential leads to higher stomatal conductance and thus transpiration. The duration of the day for which T&C‐HC predicts higher water potentials compared with T&C‐H is greatly dependent on the leaf and xylem stored water that refills during night‐time. The larger the capacitance is, the higher the leaf water potential is for most of the morning hours in T&C‐HC compared with T&C‐H. This can be shown in Figure 3, where in Br‐CAX, a site with high stem water capacitance, the simulated $\psi_{l}$ in T&C‐HC is significantly higher than in T&C‐H.

Overall, in the T&C‐HC simulations, the higher leaf water potential increases transpiration without proportionally increasing GPP (Figures S3–S6). This leads to a reduced simulated water use efficiency. The reason for the reduced water use efficiency is that in the early morning high stomatal conductance increases transpiration but without an increase in carbon assimilation, as both radiation and temperature are below optimal. The water use efficiency, defined as the ratio of daily GPP to daily transpiration before drought onset, was −12.81 ± 16.7% lower in T&C‐HC than in T&C‐H for E1a and −12.2 ± 17.3 for E1b. This shows that whether drought developed under high or low atmospheric water demand had a minor impact on this variable. The differences were greatest for the GF‐GUY tropical site, which has the highest xylem water capacitance. This lower water use efficiency leads to earlier depletion of available soil water and thus earlier stress onset. The sensitivity of water use efficiency to plant water capacitance also manifests in our parameter sensitivity analysis (Figure S8), where high values of water capacitance for the Fr‐Pue site during the E1a experiment lead to higher transpiration. This high transpiration without simultaneous proportional increase in photosynthesis ultimately depletes soil in a plant inefficient way, as GPP is not equally increased.

The manner leaf water potential evolves differently in T&C‐H and T&C‐HC gives rise to distinct signatures on the diurnal patterns of GPP and transpiration. Before drought onset (Figure 2b,e), T&C‐H and T&C‐HC simulated a more pronounced decline of both GPP and transpiration in the late afternoon compared with the original model T&C. This decline is due to a declining leaf water potential during the afternoon hours (Figure 3a,d), which leads to higher stomatal closure than T&C. This suggests that the Leuning model, which depends on VPD alone, cannot properly track the behaviour of a declining leaf water potential during the day, even when VPD peaks in the late afternoon hours (Figure 3c,f). Under severe soil water limitations (Figure 2c,f), this discrepancy is augmented in relative terms, but the overall fluxes are very small to have any major difference once the drought has fully developed.

### 3.2 Drought relief

**FIGURE 4:**
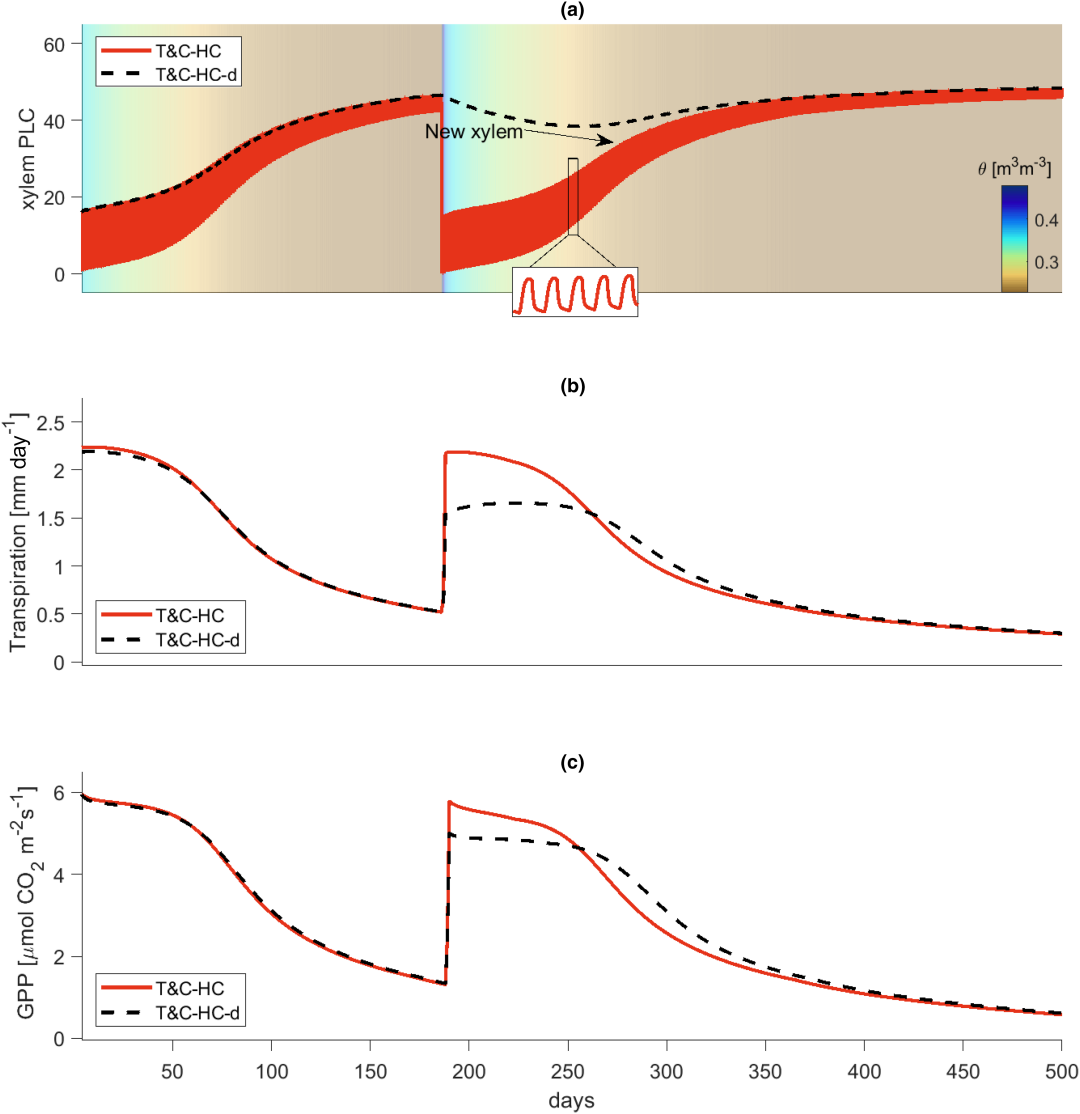
(a) Simulated percentage loss of xylem conductivity using the T&C‐HC (red) and T&C‐HC‐d (dashed black) models for Fr‐PUE. Background colours show the average soil moisture of the root zone for the dry‐down and rewatering experiments. The insert shows the daily recovery of xylem conductivity when no damage is considered in the T&C‐HC model. (b) Simulated daily plant transpiration using the T&C‐HC (red) and T&C‐HC‐d (dashed black) models for Fr‐PUE. (c) Simulated daily GPP using the T&C‐HC (red) and T&C‐HC‐d (dashed black) models for Fr‐PUE.

Looking at ecosystem recovery post major droughts, irreparable loss of conductivity in the xylem plays a critical role (Figure 4). As droughts develop, the loss of xylem conductivity predicted by the versions T&C‐HC and T&C‐HC‐d is almost identical, with the only exception being the restoration of conductivity during night‐time predicted by T&C‐HC (Figure 4a, insert). This restoration is not possible in T&C‐HC‐d as lost conductivity is only recovered by building new xylem. However, given the almost identical strength of conductivity loss between T&C‐HC and T&C‐HC‐d during the daytime, simulated water and carbon fluxes were almost identical between the two representations (Figure 4b,c) during the first dry‐down. After soil rewetting, however, simulations between T&C‐HC and T&C‐HC‐d diverge (Figure 4b,c). When permanent damage of the xylem is considered in the model T&C‐HC‐d, photosynthetic rates and transpiration were lower postrelief compared with the model that assumed immediate recovery of conductivity (T&C‐HC). Immediately postrelief GPP and transpiration were lower by −14.2 ± 16.0% and −23.9 ± 41.6%, respectively, when xylem damage was considered under (E2a). Under (E2b) the postrelief, differences were almost identical to E2a, that is −10.3 ± 11.3% and −23.9 ± 40.7% for GPP and transpiration, respectively, indicating that the strength of atmospheric drought also has little impact on the recovery dynamics.

This numerical result highlights the productivity drought legacy following major droughts (e.g. Müller & Bahn, 2022). Recovery was not slower only for the fluxes but also for the recovery of leaf area. Postdrought, the leaf area index (LAI) in T&C‐HC‐d recovers at a slower rate (Figure S9). These are interlinked, as low carbon gross (and net) primary productivity postdrought decelerated the recovery of LAI. This lag of LAI recovery in T&C‐HC‐d also caused a delay in stress onset in subsequent droughts (Figure 4b). Low LAI combined with low xylem conductivity reduced plant transpiration during recursive droughts, depleting soil water stores at a slower pace and thus leading to a delay on stress onset. However, this behaviour occurred only after very prolonged droughts capable of inducing considerably high damage to the xylem. Droughts of this length were rare in the meteorological data in all of the sites used in this study.

### 3.3 Plant hydraulics, meteorological variability and ecosystem functioning

Idealized examples showed that plant hydraulics, and xylem damage legacies in ecosystem modelling lead to distinct signatures in water and carbon dynamics. It is important to understand how these signatures propagate into ecosystem response when a realistic variability of the meteorological forcing is taken into account and whether they can reproduce observed data. The questions we ask here are, (a) can we identify the simulated signatures in observations? and (b) do those signatures we observed in idealized numerical experiments play a major role, or are they masked when realistic weather forcing is used as a driver? For this reason, we analyse the overall water and carbon dynamics when all model representations were driven by observed weather data for all sites (E3).

The three distinct model signatures that were identified in the idealized numerical experiments during periods of hydrological and meteorological drought were as follows: (i) the slower productivity and transpiration reduction in the plant hydraulic models (T&C‐H and T&C‐HC) when soil droughts develop, (ii) the simulated afternoon productivity decline in carbon and water fluxes simulated by T&C‐H and T&C‐HC and (iii) the productivity legacy decline following major droughts, simulated by the models that include long‐term xylem damage (T&C‐HC‐d).

**FIGURE 5:**
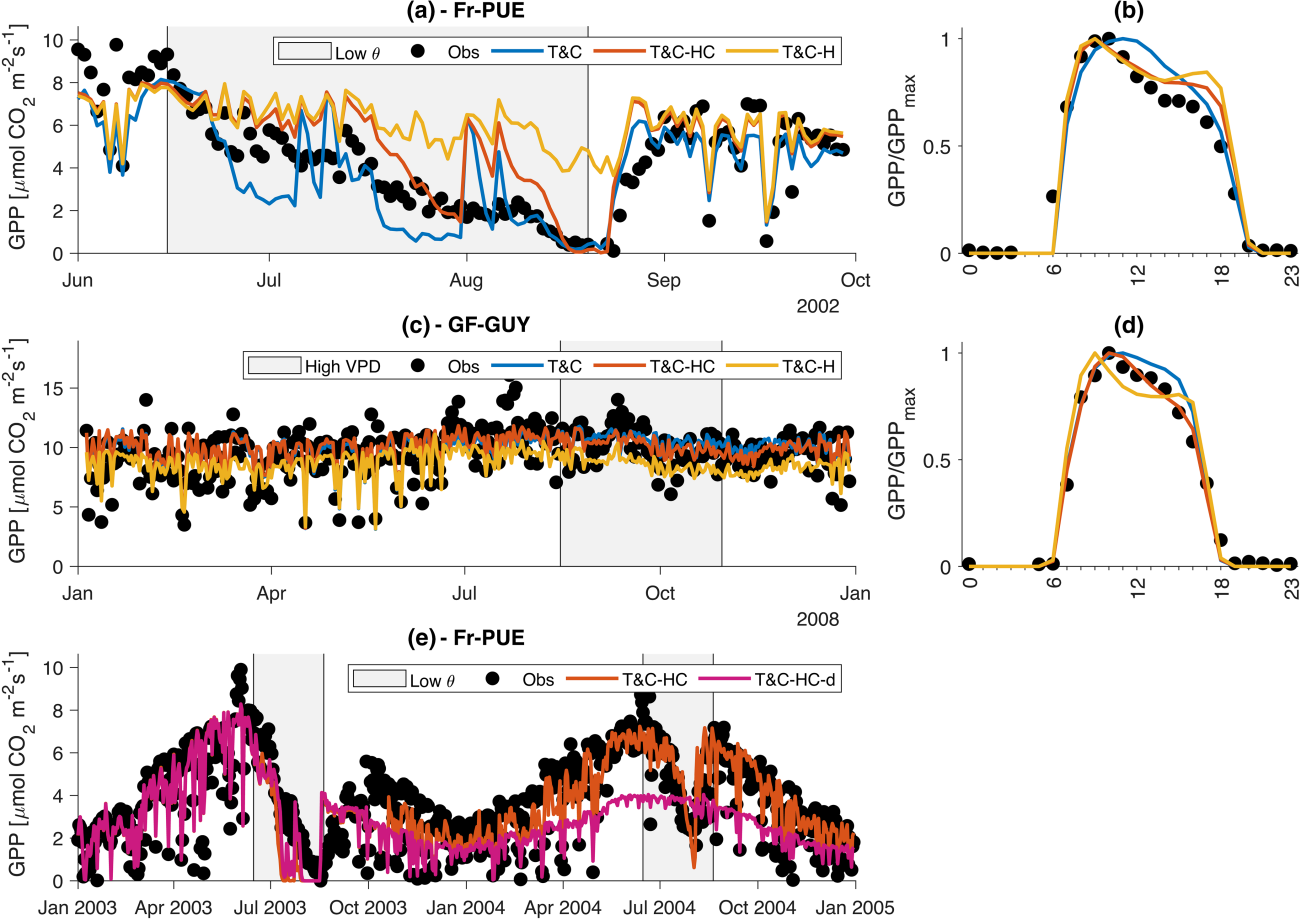
(a) Observed and simulated using T&C, T&C‐H and T&C‐HC daily gross primary productivity for the Fr‐Pue site during the summer–fall of 2022. (b) Observed and simulated average diurnal distribution of gross primary productivity for the Fr‐Pue site during the drought period 15 June to 20 August 2002. (c) Same as (a) but for the GF‐GUY site. (d) Same as (b) but for the GF‐GUY site during the period 15 August to 30 October 2008 when high atmospheric water demand (high vapour pressure deficit) occurs. (e) Save as (a) but for the models T&C‐HC and T&C‐HC‐d during 2003–2004.

Looking at a site that experiences seasonal intense soil moisture limitations (Fr‐PUE), during a typical year (2002) (Figure 5a; Figure S14), we observed, as expected from the idealized experiments, a slower decline in productivity simulated by T&C‐H and T&C‐HC compared with T&C. To some degree, this is compatible with observations. For example, during the period 15 June to 25 June, T&C simulated a very sharp productivity decline, from 8 to 2 μmol CO_2_ m^−2^ s^−1^ which is much faster than the observed decline from 8 to 4 μmol CO_2_ m^−2^ s^−1^. In general, the very abrupt productivity decline rates simulated with T&C are not observed showing the potential of T&C‐H and T&C‐HC to provide better results, if the parameters of xylem and leaf vulnerability were better known. However, because there was no model calibration tuned to reproduce this exact behaviour, T&C‐H and T&C‐HC in Fr‐PUE underestimated the overall decline.

In terms of diurnal patterns, T&C‐H and T&C‐HC outperform significantly T&C during both hydrological (Figure 5b) and atmospheric drought (Figure 5d). The observed afternoon decline in productivity could only be reliably reproduced when plant hydraulic models were used. Moreover, in the tropical site GF‐GUY, where plant water storage is the highest among all simulations, T&C‐HC outperformed all other models, showing the importance of introducing capacitance in those ecosystems.

In Figure 5e, we show the simulated drought legacy that T&C‐HC‐d produced following the major 2003 heatwave, which led to large negative soil moisture anomalies in Fr‐PUE. Our parameterization of xylem damage and repair significantly overestimated drought legacies during 2004, with T&C‐HC‐d giving considerably worse results than the model that did not include xylem damage. This is in line with the findings of Page et al. (2023) who showed that drought effects and their ensuing feedbacks on fluxes are rare beyond 6‐month postdrought. The main reason for this result is that no change in the carbon allocation rules, conditional to xylem damage, was added to the T&C‐HC‐d model. This likely underestimated the plant's prioritization of restoring functional xylem tissues post a major drought leading to unrealistically high legacies. Consequently, the model variants that included xylem damage yielded worse results in all sites.

**FIGURE 6:**
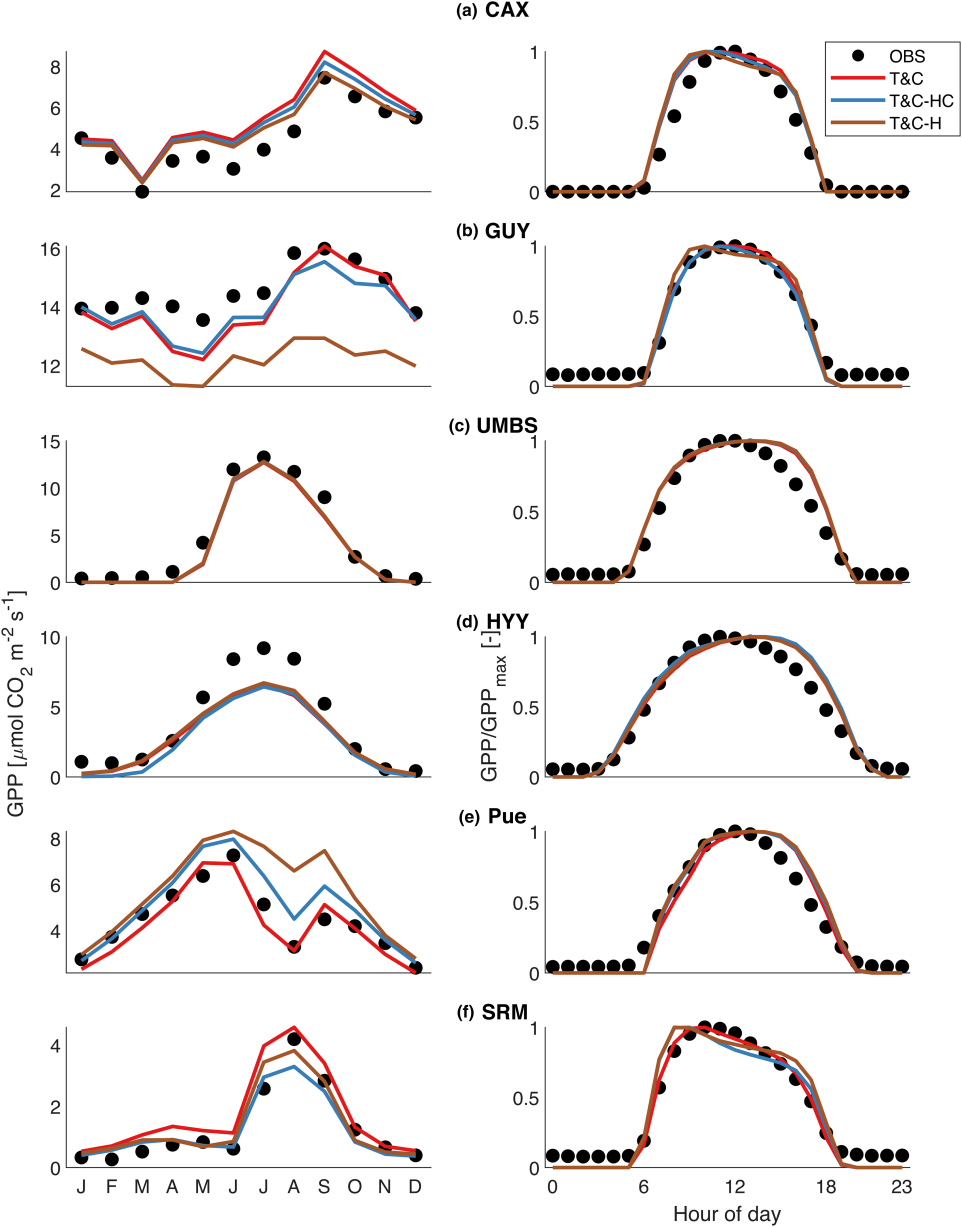
Left: observed and simulated gross primary productivity for (a) Br‐CAX, (b) GF‐GUY, (c) US‐UMB, (d) FI‐HYY, (e) Fr‐Pue and (f) US‐SRM. Normalized diurnal variability of gross primary productivity for the three most active months.

**FIGURE 7:**
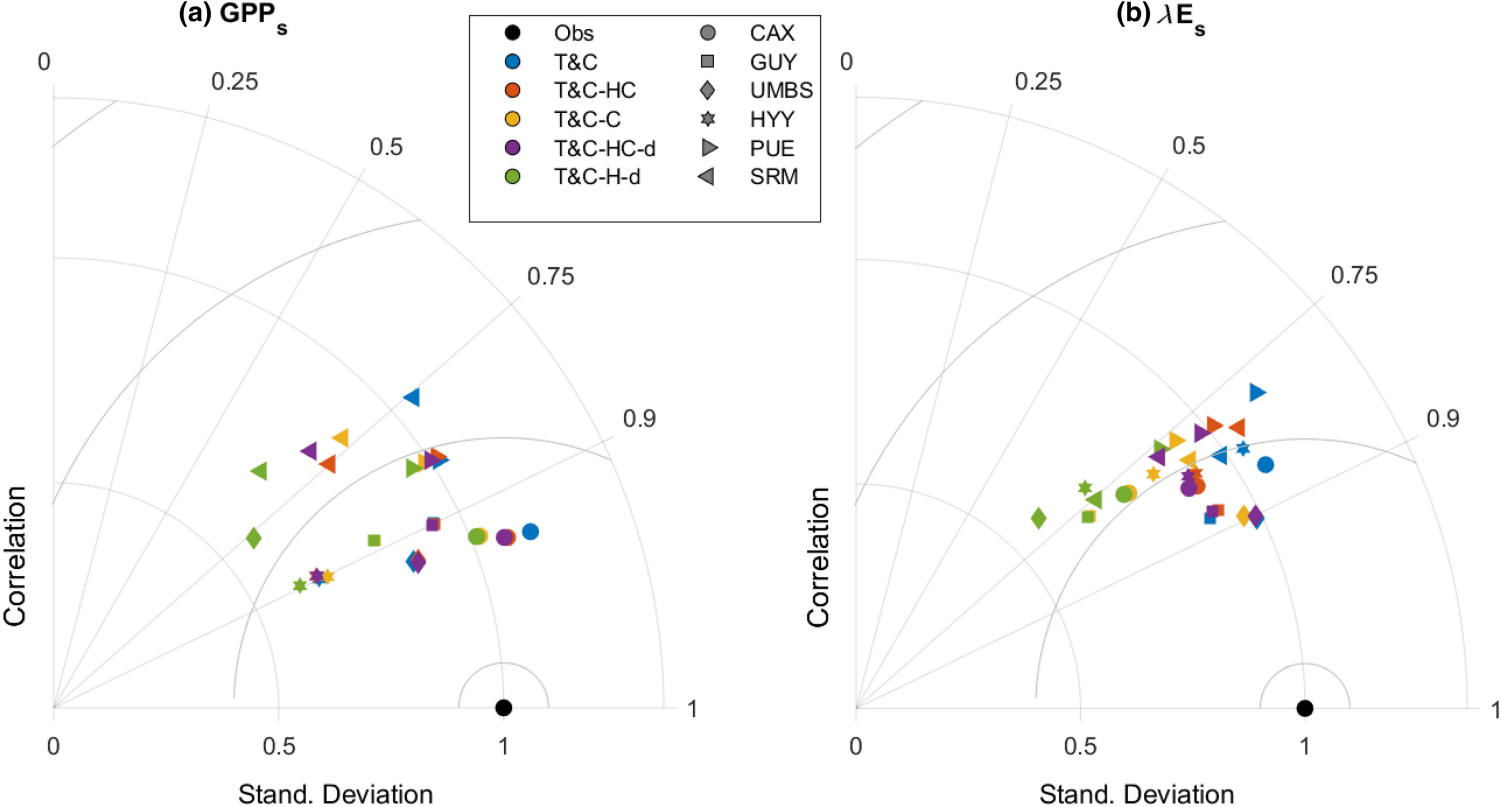
Taylor diagrams for all sites and models for daily gross primary productivity (a) and daily latent heat fluxes (b). For direct across‐site comparison, observed and simulated fluxes were normalized by dividing them with the standard deviation of the observed fluxes for each site.

Looking beyond stress periods, at the overall seasonal and diurnal patterns of GPP and evapotranspiration (Figure 6; Figure S7), as well as model validation statistics (Table 2; Figure 7) all model variants perform similarly. This result highlights that when plant hydraulics are introduced, even though under stress periods they can significantly improve model results, they are not adequate to provide an overall considerable model performance boost. In fact, in some cases the results are marginally worse for T&C‐HC and T&C‐H compared with the default T&C (Table 2; Figure 7). The main reason for this is the identical parameterization of $f_{s}$ among models which favoured T&C, as the parameters were originally obtained for default T&C simulations. Part of the discrepancy is also due to our choice to avoid automated calibration of the vegetation hydraulic properties and rely when possible on published parameters instead. What is of major importance is that the model‐observation differences far exceed the model‐to‐model differences. The average monthly GPP model‐data absolute difference for all stations is 2.3, 2.2, 2.3 μmol CO_2_ m^−2^ s^−1^ for T&C, T&C‐HC and T&C‐H, respectively. Average monthly intermodel range for all stations is just 0.7 μmol CO_2_ m^−2^ s^−1^.

**TABLE 2:** Coefficient of determination *r* ^2^, root mean square error (RMSE) and Kling–Gupta Efficiency (KGE) for gross primary productivity (GPP), latent heat *λE* and sensible heat flux *H*, estimated between observations and simulations at the hourly time scale.

	GPP					*E*					*H*				
	T&C	T&C‐HC	T&C‐H	T&C‐HC‐d	T&C‐H‐d	T&C	T&C‐HC	T&C‐H	T&C‐HC‐d	T&C‐H‐d	T&C	T&C‐HC	T&C‐H	T&C‐HC‐d	T&C‐H‐d
	*r* ^2^ [‐]					*r* ^2^ [‐]					*r* ^2^ [‐]				
Br‐CAX	.88	.88	.86	.87	.86	.74	.70	.62	.70	.61	.50	.58	.61	.58	.61
GF‐GUY	.81	.81	.79	.81	.79	.78	.77	.60	.76	.59	.73	.71	.76	.71	.76
US‐UMB	.86	.86	.86	.86	.58	.82	.81	.80	.81	.48	.79	.79	.79	.79	.71
FI‐Hyy	.81	.80	.81	.80	.80	.69	.68	.62	.67	.52	.64	.65	.66	.66	.66
Fr‐PUE	.71	.73	.73	.53	.23	.65	.62	.60	.51	.49	.77	.75	.76	.75	.74
US‐SRM	.57	.56	.53	.50	.43	.68	.65	.64	.59	.57	.91	.90	.91	.90	.90

	RMSE [μmol m^−2^ s^−1^]					RMSE [W m^−2^]					RMSE [W m^−2^]				
Br‐CAX	3.51	3.30	3.29	3.29	3.29	83.39	76.70	84.03	76.83	84.55	49.55	45.59	54.13	45.91	55.10
GF‐GUY	5.68	5.64	6.14	5.63	6.16	70.95	71.99	98.57	72.52	99.19	37.89	41.66	71.06	42.38	71.66
US‐UMB	2.80	2.79	2.75	2.76	5.13	32.94	33.40	33.91	33.34	56.12	47.78	47.94	49.33	48.00	74.37
FI‐Hyy	2.76	2.86	2.68	2.85	2.93	27.91	26.65	28.64	26.71	32.12	49.72	50.34	51.92	50.47	55.91
Fr‐PUE	2.62	2.82	3.15	3.40	4.62	35.80	35.27	34.15	33.39	34.26	55.74	59.73	59.50	65.53	66.40
US‐SRM	1.57	1.37	1.46	1.49	1.55	26.75	28.90	27.45	29.13	29.79	56.69	55.37	58.32	56.19	59.49

	KGE [‐]					KGE [‐]					KGE [‐]				
Br‐CAX	0.74	0.79	0.83	0.79	0.83	0.49	0.63	0.64	0.64	0.64	0.53	0.49	0.17	0.46	0.13
GF‐GUY	0.87	0.87	0.82	0.87	0.81	0.80	0.85	0.66	0.84	0.65	0.85	0.72	−0.34	0.67	−0.38
US‐UMB	0.86	0.87	0.87	0.87	0.49	0.84	0.86	0.85	0.86	0.53	0.52	0.50	0.49	0.50	0.03
FI‐Hyy	0.71	0.68	0.73	0.68	0.67	0.69	0.77	0.72	0.76	0.64	0.67	0.47	0.43	0.45	0.22
Fr‐PUE	0.84	0.74	0.60	0.55	0.05	0.59	0.51	0.63	0.64	0.61	0.24	0.30	0.18	−0.14	−0.25
US‐SRM	0.10	0.51	0.38	0.29	0.33	0.80	0.80	0.79	0.73	0.67	0.74	0.77	0.74	0.77	0.71

Looking at model performance at the daily time scale, for all sites (Table 2; Figure 7) the introduction of plant hydraulics does not change significantly model performance. All model variants had an almost identical correlation coefficient between daily observations and simulations. Simulated variances in both water and carbon fluxes (Figure 7) declined when plant hydraulics are used. A major significant difference worth mentioning occurred for the temperate site UMBS, where the model variant with hydraulics and irreversible conductivity loss (T&C‐H‐d) predicted a much lower variability in the fluxes than in the observations, and its overall performance was substantially lower than the rest of the model variants. The reason for this behaviour was that during winter UMBS experiences soil freezing (Figure S13). When soil freezes, the water potential in the soil drops significantly following the freeze–thaw dynamics introduced in T&C (Yu et al., 2020). In the model formulation of T&C‐H‐d, when deciduous vegetation is present, during dormant periods where there are no leaves, the solution of the system of Equations (3) and (4) leads to $\psi_{x}=\psi_{s}$ to result in a zero transpiration flux. That leads to a major loss of conductivity that cannot be restored when soil thaws and water potential increases. This is not the case when xylem water storage is included as in our model formulation we do not allow water movement from the xylem to the soils (i.e. hydraulic redistribution). This means that during very low soil water potentials when soil freezes, the high xylem water content sustains a high xylem water potential that does not lead to major conductivity losses. We refrain from further interpretation of this result as it is not realistic to have major damages in response to soil freezing, but it is an important warning for plant hydraulic formulations aimed at long‐term simulations in cold climates. This behaviour could be numerically avoided if the soil‐to‐root conductivity was set to zero during freezing periods, or if the soil freezing module was disabled. However, it is important to mention that introduction of long‐term damage in both the T&C‐HC‐d and T&C‐H‐d leads to worse results compared with all other model variants, as clearly shown for the Fr‐PUE site (Figure 5e).

**FIGURE 8:**
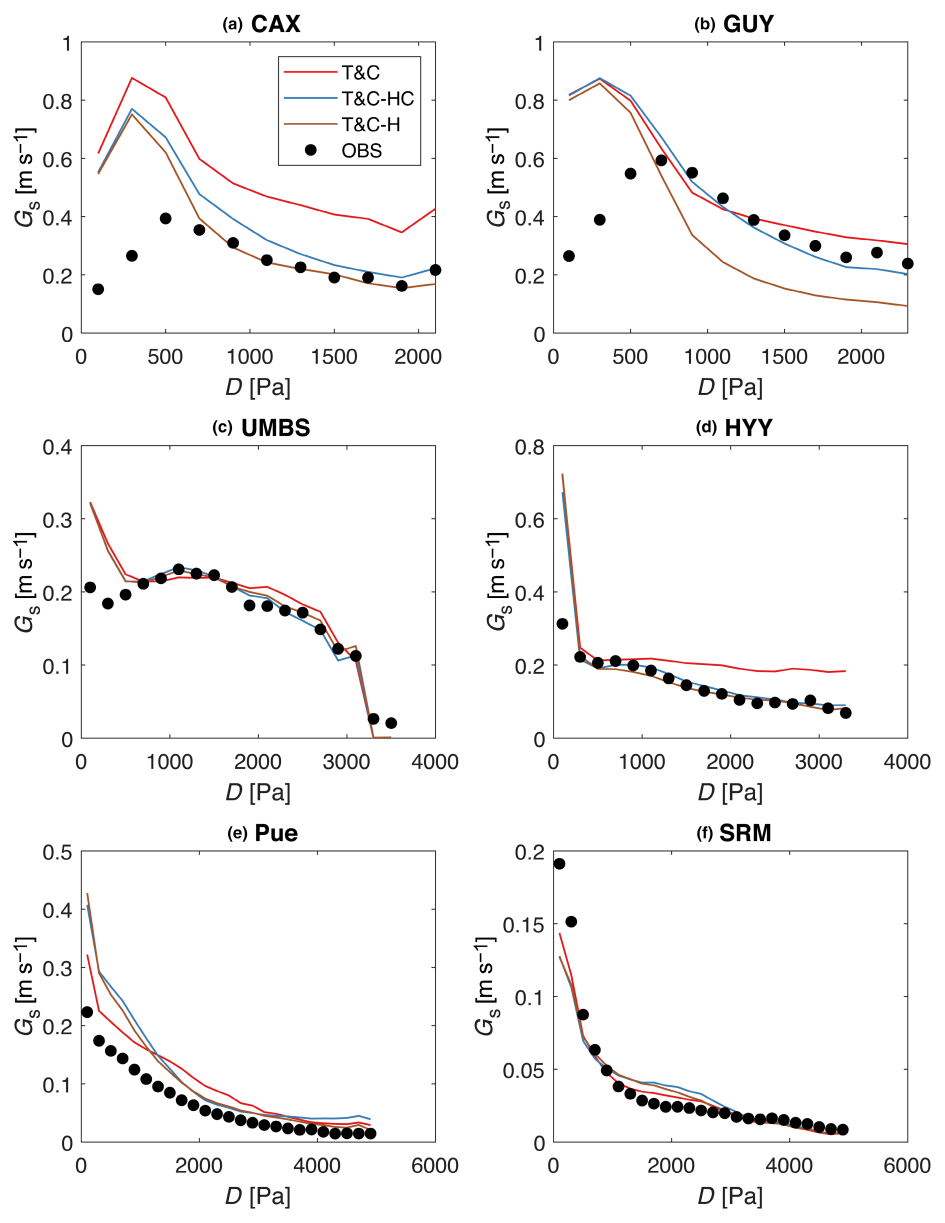
Estimated average surface conductance for bins of a 200 Pa width computed by inverting the Penman–Monteith equation for eddy covariance observations (dots) and model simulations (lines).

The response of the ecosystem bulk surface conductance to atmospheric dryness (i.e. VPD) was similar between all model variants (Figure 8). Introducing plant hydraulics had some marginal improvement regarding the sensitivity of surface conductance to VPD, but the overall patterns were similar across all models. Model performance was better for both T&C‐H and T&C‐HC in Br‐CAX, Fr‐Pue and FI‐HYY. In GF‐GUY, T&C‐HC improved the simulations marginally, but T&C‐H made them worse, showing the importance of introducing capacitance in forests where trees can store substantial amounts of water. In both tropical sites, all variants overestimated surface conductance at very low VPD values (Figure 8a,b). Low observed surface conductance at low values of VPD however could be related to observational uncertainty with conditions that are typically associated with night‐time stable atmospheric profiles or rainy conditions, difficult to observe with flux towers. The sensitivity of stomatal conductance to environmental forcing using the simple Leuning formulation was adequate enough to even capture salient features of the water fluxes, such as the hysteretical pattern between evapotranspiration and VPD during the day (Figure S11), a behaviour that has been partially attributed to plant hydraulics before (Mirfenderesgi et al., 2016).

**FIGURE 9:**
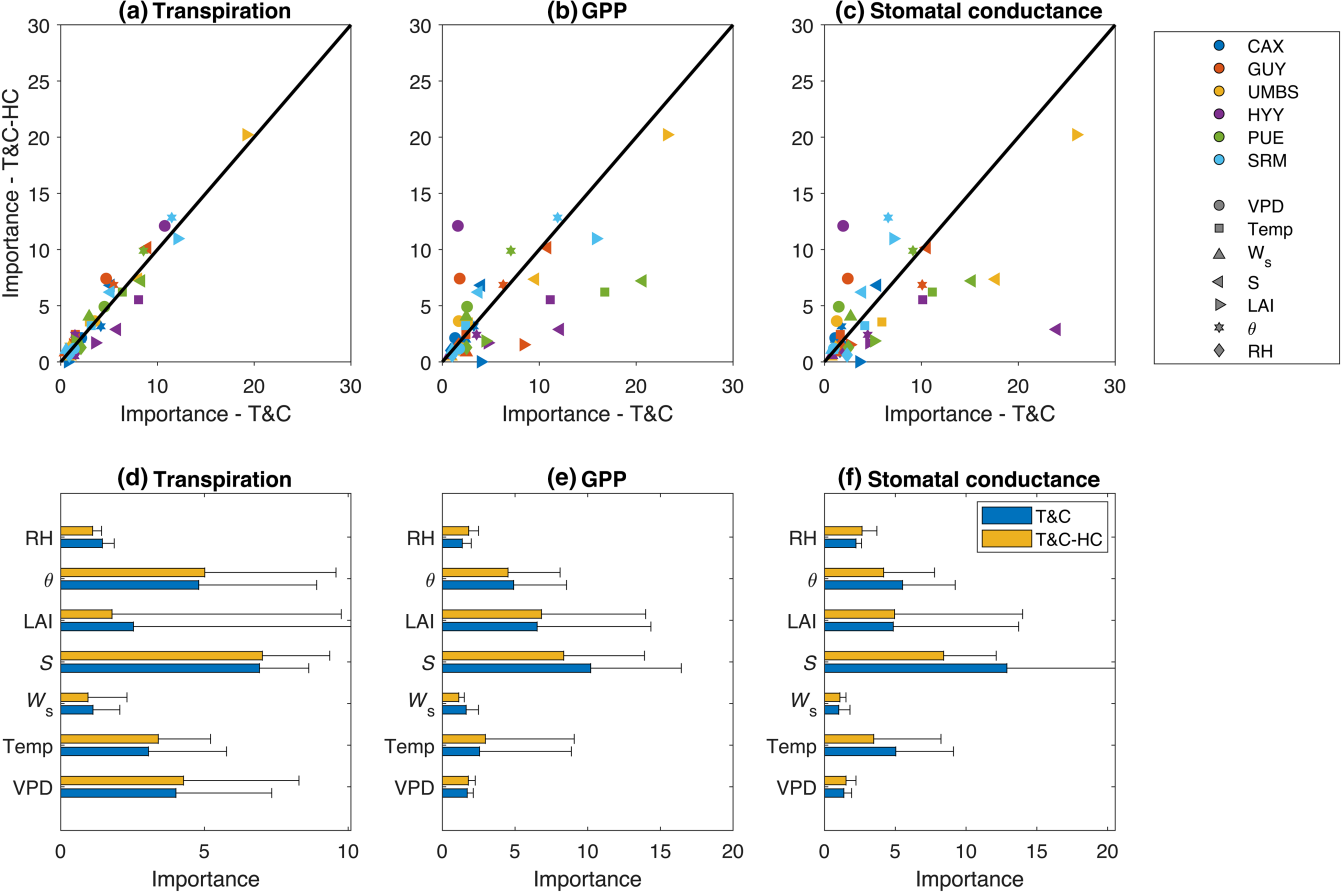
Scatter plots of predictor importance estimates by permutation of out‐of‐bag predictor observations for a random forest of regression tree between T&C and T&C‐HC for (a) daily transpiration (b) daily gross primary productivity and (c) daily average stomatal conductance. (d–f) Error bars showing the average predictor importance values across all sites (bar height) and their standard deviations (whisker height), for all predictors.

Running an attribution analysis for all model variants, different aspects of environmental forcing (radiation, soil moisture, relative humidity, temperature, wind speed and VPD) and ecosystem structure (LAI) explained to a similar degree the dynamics of stomatal conductance that influence transpiration and photosynthesis (Figure 9). The same results were also reproduced when Shapley values were used for feature attribution (Figure S12).

Radiation explained most of the variability of the stomatal conductance for all models (Figure 9f). The reason is that in all model formulations, stomatal conductance is proportional to net leaf photosynthesis, which is highly impacted by absorbed radiation. This dependence is the same for all models, as they share the same canopy radiation transmission scheme and photosynthesis biochemical model. Differences due to the introduction of plant hydraulics are expected to occur due to different stomatal responses to soil moisture and VPD. However, those two variables explain a smaller fraction of the variability of stomatal dynamics compared with radiation, with atmospheric humidity playing a minor role compared with soil moisture, ultimately leading to small differences between the simulated stomatal conductance dynamics using the different variants.

LAI also explains a large fraction of the variability of GPP (Figure 9e), similar in magnitude to soil moisture. This is not unexpected as GPP scales with the available leaf area. Overall, radiation and LAI explain a large fraction of the variability of GPP. As all models share the same dynamic vegetation component (i.e. allocation of assimilated carbon in different carbon pools and phenology), the simulated LAI is very similar, leading to small differences in GPP between the model formulations used in this study.

Regarding plant transpiration (Figure 9d), solar radiation, soil moisture, VPD and temperature explain most of its variability. Solar radiation affects transpiration by providing the required energy for water evaporation and by modulating stomatal conductance. As explained, previously both aspects impact all model variants similarly. VPD affects transpiration by modulating stomatal conductance and driving transpiration (i.e. $T\propto g_{s}D$). Stomatal conductance responses to VPD, shown in Figure 8, were similar across models; thus, VPD affects all models in a similar manner. It is important to mention that VPD also covaries strongly with solar radiation and temperature, and thus, part of the same responses to VPD relate to the way all models respond to changes in temperature and radiation (which is identical among all models) rather than VPD. In fact, when this covariation is weakened as in the idealized dry‐down experiments, VPD affects plant hydraulics formulations differently than in the Leuning model. That indicates that the agreement between stomatal conductance and VPD (Figure 8) between models, to a large degree, originates from the same responses to light and temperature rather than VPD itself. Additionally, the differences in the responses of stomatal conductance to VPD leading to pronounced differences in the diurnal variability of carbon and water fluxes during stress periods (Figure 5b,d) are mostly masked when the whole simulation is taken into account, as those periods cover a small fraction of time.

Finally, the formulation of all models regarding their responses to soil moisture was identical via the common stress factor $f_{s}$, and thus, the large influence of soil moisture explaining plant transpiration is expected to impact all models equally, regardless of their inclusion of plant hydraulics.

## 4 IMPLICATIONS FOR TERRESTRIAL BIOSPHERE MODEL DEVELOPMENT

A rising concern related to more common plant mortality events and increasing drought severity (e.g. Hartmann et al., 2018) combined with emerging datasets of plant hydraulic traits (e.g. Choat et al., 2012; Kattge et al., 2020; Martin‐StPaul et al., 2017) and a better knowledge of internal plant hydrodynamics (e.g. Sperry & Love, 2015) has led many to suggest that explicitly modelling plant hydraulic and considering plant hydraulic traits in terrestrial biosphere models might represent a turning point for vegetation representation in land surface models (e.g. Li et al., 2021; Matheny et al., 2017; Ruffault et al., 2022).

This idea has been corroborated by several studies that showed increased model skill when mechanistic plant hydraulics models were used. For instance, successful plant hydraulic implementations have been introduced to the widely used models ED2 (Xu et al., 2016), CLM (Kennedy et al., 2019), ORCHIDEE (Naudts et al., 2015) and NOAH‐MP (Li et al., 2021), among others. Introduction of plant hydraulics into terrestrial biosphere models has shown significant improvements in terms of carbon and water fluxes in several sites across the world, including the sites we report in this paper (e.g. Kennedy et al., 2019).

The results we present in this study, generally representative of longer periods, moderate this enthusiastic view. We show that introducing plant hydraulics in a terrestrial biosphere model through a stress factor applied stomatal conductance can improve model performance during periods of stress, leading to more realistic diurnal patterns of carbon and water fluxes, and a better representation of stress onset. However, the unique realistic model signatures introduced by plant hydraulics get largely masked by climate variability. This may only lead to a marginal improvement on long‐term water and carbon dynamics, all other things being equal. Note, however, that models which, instead of relying on a hydraulics‐driven stress factor ($f_{s}$ and $f_{l}$ here; see Section 2) to downregulate stomatal conductance, optimize stomatal function depending on a combination of photosynthetic and hydraulic processes might lead to more important improvements on long‐term water and carbon dynamics (e.g. Sabot, De Kauwe, Pitman, Ellsworth, et al., 2022; Sabot, De Kauwe, Pitman, Medlyn, et al., 2022). Regardless, improving model realism during stress gives rise to new modelling opportunities, particularly on how to link plant hydraulics to the remaining ecosystem processes. A crucial example would be to link plant hydraulics with plant phenology. Xu et al. (2016) showed that when linking hydraulics to phenology within the ED2 model, simulation results improved significantly. Several recent data‐driven modelling studies further support that leaf area dynamics during drought clearly relate to plant tissue exposure to drought (e.g. Nadal‐Sala et al., 2021; Nadal‐Sala et al., 2023; Sabot, De Kauwe, Pitman, Ellsworth, et al., 2022; Sabot, De Kauwe, Pitman, Medlyn, et al., 2022). Additionally, the inclusion of plant hydraulics, enabling the quantification of water potentials across plant tissues, provides a clear opportunity linking plant hydraulics to hydraulic failure and plant mortality, which is not considered in this study.

Looking in more detail at the unique model signatures during drought development, we identified the important role of plant water storage, in agreement with previous studies (e.g. Hartzell et al., 2017; Huang et al., 2017). Plant water stores were found to highly modulate the way plants respond to atmospheric water demand by altering their water use efficiency. This suggests that the introduction of plant water capacitance in ecosystem modelling is important, particularly in ecosystems where plants can rely for long periods of time on internal water resources. This represents a major challenge considering that plant traits linked to plant water storage are rarely available at the species level, let alone at the ecosystem scale. The fact that plant hydraulic models are particularly sensitive to those exact parameters calls for extensive global‐scale data collection.

In agreement with Liu et al. (2020), we found that when plant hydraulics are neglected, soil moisture plays a disproportionately high role in determining stomatal conductance and thus water and carbon fluxes. This can be illustrated with the much more abrupt stress onset as soils dry when empirical stomatal conductance models are used. This higher sensitivity leading to faster stress onset can be crucial under climate change, as the temporal structure of rainfall is expected to change (Moustakis et al., 2022; Ukkola et al., 2020). That could potentially lead to an overestimation of the importance of hydrological droughts when simple model structures are used; a crucial problem considering that state‐of‐the‐art terrestrial biosphere models already struggle to accurately simulate ecosystem responses to drought (e.g. Paschalis et al., 2020; Powell et al., 2013).

We showed that the impairment of the conducting system in plants can only be accurately simulated when plant hydraulics are properly introduced in terrestrial biosphere models. This can impact postdrought ecosystem recovery. Previous studies showed that when plant mortality is linked to hydraulics failure in terrestrial biosphere models (e.g. Yao et al., 2022), their performance improves significantly. Our results show that if impairment of the xylem is considered when resolving plant hydraulics, postdrought recovery can be considerably slower following prolonged dry periods. This could partially resolve the weakness of current generation models, not being capable of capturing long‐term drought legacies (Anderegg et al., 2015). However, we clearly illustrated that if impairment is to be considered, we need new dynamic vegetation modules that consider flexible carbon allocation patterns based on the level of xylem damage. This is an additional opportunity to link plant hydraulics with other ecosystem processes. This calls for tailored hydrological and atmospheric drought experiments at the ecosystem scale to obtain the data needed to develop new carbon allocation schemes, explicitly linked to the hydraulic behaviour of plants.

We have to stress out that our results can be affected by how the T&C model simulates all other ecosystem processes beyond plant hydraulics. It would be highly beneficial to see similar studies, employing multiple plant hydraulic parameterizations in other ecosystem models, in order to further facilitate detailed model intercomparisons.

Finally, the level of detail needed to properly capture plant hydraulic behaviour remains challenging. In this article, we only used a simple lumped representation of plant hydraulics, which was tested in a limited number of sites. While we showed that this approximation captures dynamics such as the lag between transpiration and sap flux, and the diurnal variability of water and carbon fluxes, it still falls short of describing the full complexity of the problem. Previous studies have shown a large variability of plant hydraulic traits, across and within ecosystems (e.g. Anderegg, 2015; Garcia et al., 2022). In those cases, a lumped approximation might be inadequate, and detailed spatially explicit approaches might be needed (Bohrer et al., 2005; Mirfenderesgi et al., 2016), especially considering the high model sensitivity to hydraulic traits (Figure S8). We might also need to further refine the modelling of the complex water pathways in plants, considering their full symplastic and apoplastic pathways (e.g. Scoffoni et al., 2023). However, to parameterize plant hydraulic processes, either in a lumped or spatial explicit way, detailed data regarding those plant hydraulic traits are needed, which are currently not readily available. As modellers, it is important not to leapfrog observational evidence (Feng, 2020). Initiatives such as TRY (Kattge et al., 2020) are helping unify data collection, and data protocols to achieve this, but we still need a much wider global coverage of plant hydraulic data to support model parameterizations in reliably in any relevant biome. Remote sensing could further facilitate this task as recent studies have shown that crucial plant hydraulic properties can be computed by satellite sensors (Holtzman et al., 2021; Liu et al., 2021; Zhao et al., 2022).

## AUTHOR CONTRIBUTIONS

**Athanasios Paschalis:** Conceptualization; data curation; formal analysis; methodology; software; visualization; writing – original draft; writing – review and editing. **Martin G. De Kauwe:** Conceptualization; methodology; writing – review and editing. **Manon Sabot:** Conceptualization; methodology; writing – review and editing. **Simone Fatichi:** Conceptualization; methodology; software; writing – review and editing.

## CONFLICT OF INTEREST STATEMENT

The authors declare no conflict of interest.

## Supporting information


Data S1.

## Data Availability

The PLUMBER2 meteorological data used in this study can be accessed here: https://researchdata.edu.au/plumber2‐forcing‐evaluation‐surface‐models/1656048. The sap flow measurements from SAPFLUXNET used in this study can be accessed here: https://zenodo.org/records/3971689. The model code and output data that support the findings of this study are openly available in from Zenodo at https://zenodo.org/records/10039621 (http://doi.org/10.5281/zenodo.10039621).
