# Supplementary material for: When do plant hydraulics matter in terrestrial biosphere modelling?
Source: Glob Chang Biol. 2023 Nov 14;30(1):e17022. doi: 10.1111/gcb.17022 (PMC10952296; doi:10.1111/gcb.17022)
Supplement: Supplementary file 1 — Data S1. [file GCB-30-0-s001.zip › Supplementary table captions.docx]

Table S1: Caption

Model parameters and parameter sources for each of the sites.
