# Supplementary material for: When do plant hydraulics matter in terrestrial biosphere modelling?
Source: Glob Chang Biol. 2023 Nov 14;30(1):e17022. doi: 10.1111/gcb.17022 (PMC10952296; doi:10.1111/gcb.17022)
Supplement: Supplementary file 1 — Data S1. [file GCB-30-0-s001.zip › SupplMaterial.pdf]

Supplementary Material: When do plant hydraulics matter in terrestrial biosphere modelling?

Athanasios Paschalis[1], Martin G. De Kauwe[2], Manon Sabot[3], and Simone Fatichi[4]

[1]Department of Civil and Environmental Engineering, Imperial College London, UK

[2]School of Biological Sciences, University of Bristol, Bristol, BS8 1TQ, UK

[3]Climate Change Research Centre, University of New South Wales, Sydney, NSW 2052, Australia

[4]Department of Civil and Environmental Engineering, National University of Singapore

September 19, 2023

1 Supplementary Figures and Tables

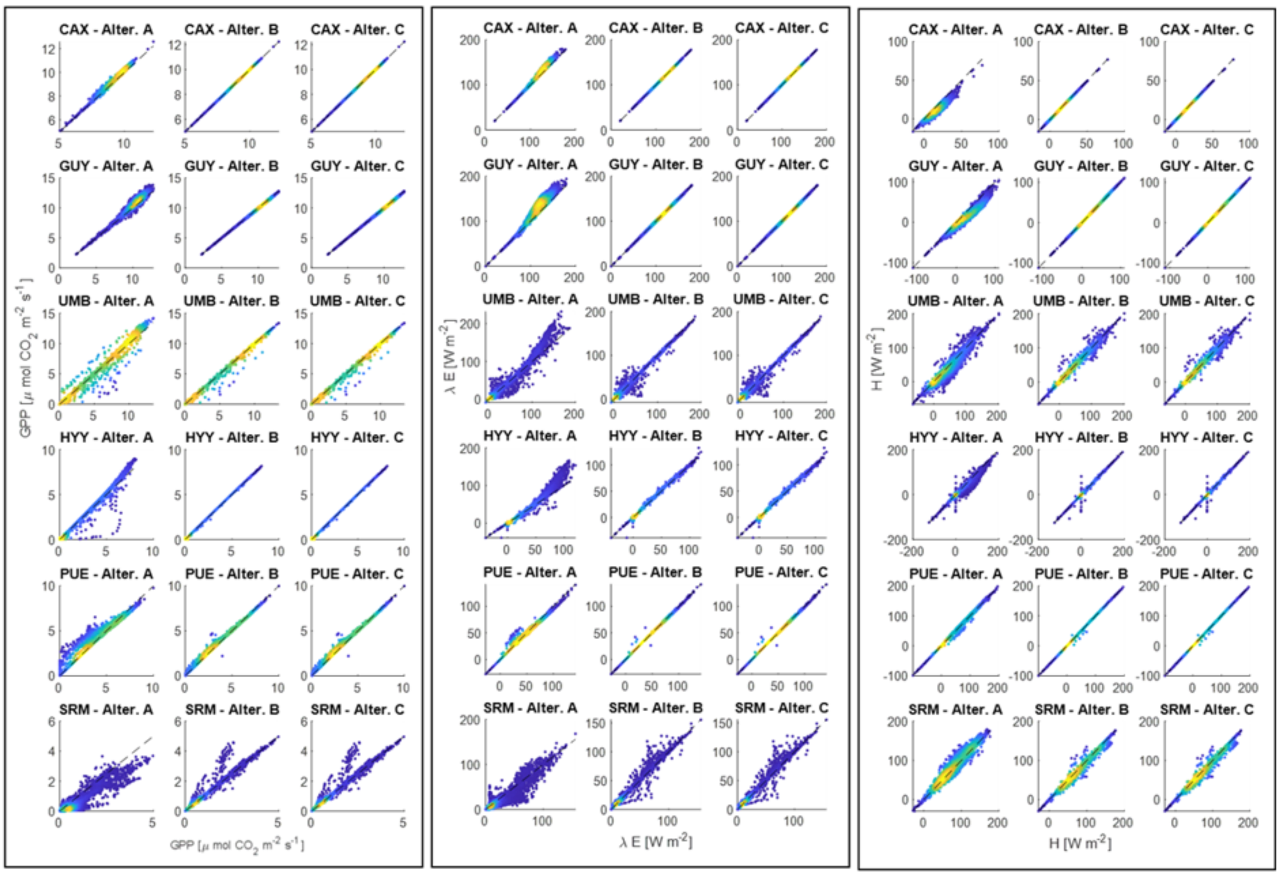

Figure S1: Scatterplots between the simulated daily average gross primary productivity GPP (left), latent heat flux λE (middle) and sensible heat flux H (right) using the parameterization of stomatal conductance we reported in the manuscript model (x-axis) and three alternative stomatal conductance models (y-axis) for all six sites. The colours of the points correspond to the probability density for each simulation. The simulations relate to the E3 experiment using the T&C-HC model variant. The three model alternatives are:

- Alternative A (no separate stomatal and "non stomatal" limitations). In this formulation the stomatal conductance was modeled as $g_s = g_0 + a_1 \frac{f(\psi_l) A_n}{c_i - \Gamma}$, where $f(\psi_l) = \exp(a_s|\psi_l|^{-\gamma})$).
- Alternative B (no dependence on c_i). In this formulation stomatal conductance was modelled with both stomatal and not stomatal limitations, but it was independent of c_i. Specifically $g_s = g_0 + a_1^* f_l(f_s A_n^{pot})$, where $a_1^* = a_1/0.7c_a$, with c_a being the atmospheric CO2 concentration. f_l, f_s, a_1 and A_n^{pot} follow the description of the manuscript. The stressed net assimilation is defined as $A_n^{stressed} = f_s A_n^{pot}$.
- Alternative C (no separate stomatal and "non stomatal" limitations and no dependence on c_i). This is the simplest formulation where $g_s = g_0 + a_1^* f(\psi_l) A_n$, where $a_1^* = a_1/0.7c_a$ and $f(\psi_l) = \exp(a_s|\psi_l|^{-\gamma})$.

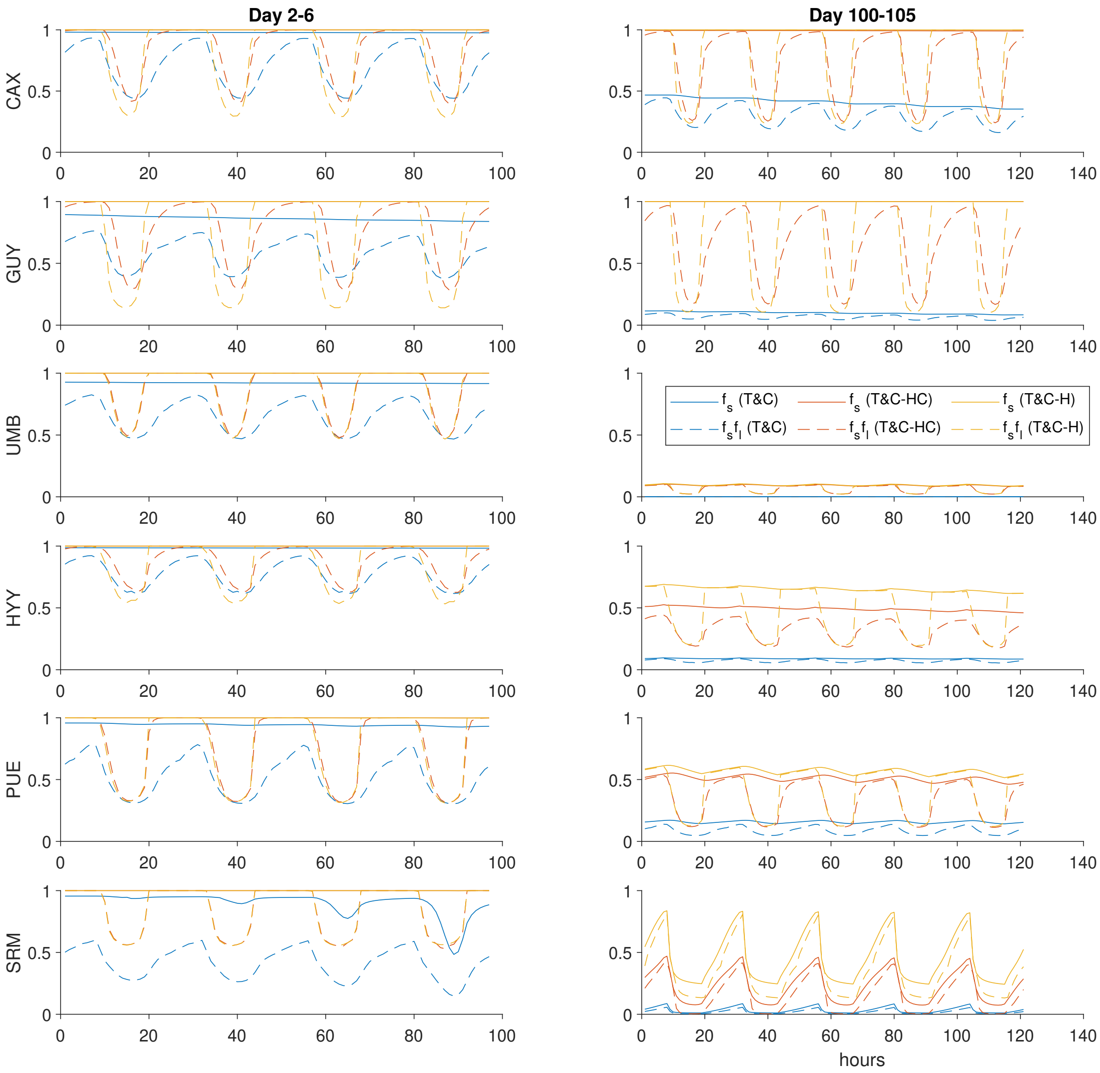

Figure S2: Simulate time series of the reduction factors f_s and $f_s * f_l$ for the dry down experiment E1b. Left columns correspond to the days 2-5 of the dry down experiment. Right columns for the days 100-105 after drought onset.

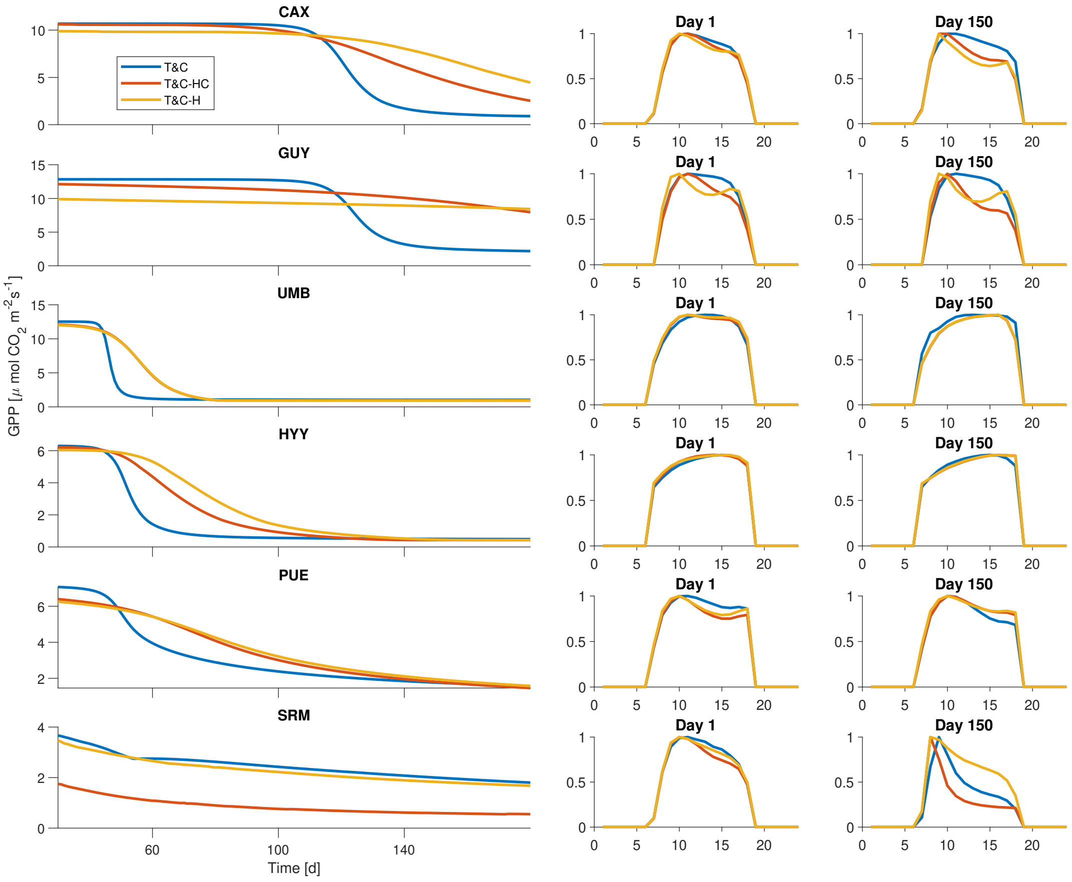

Figure S3: Same as Figure 2 in the main paper, but for all sites under E1a

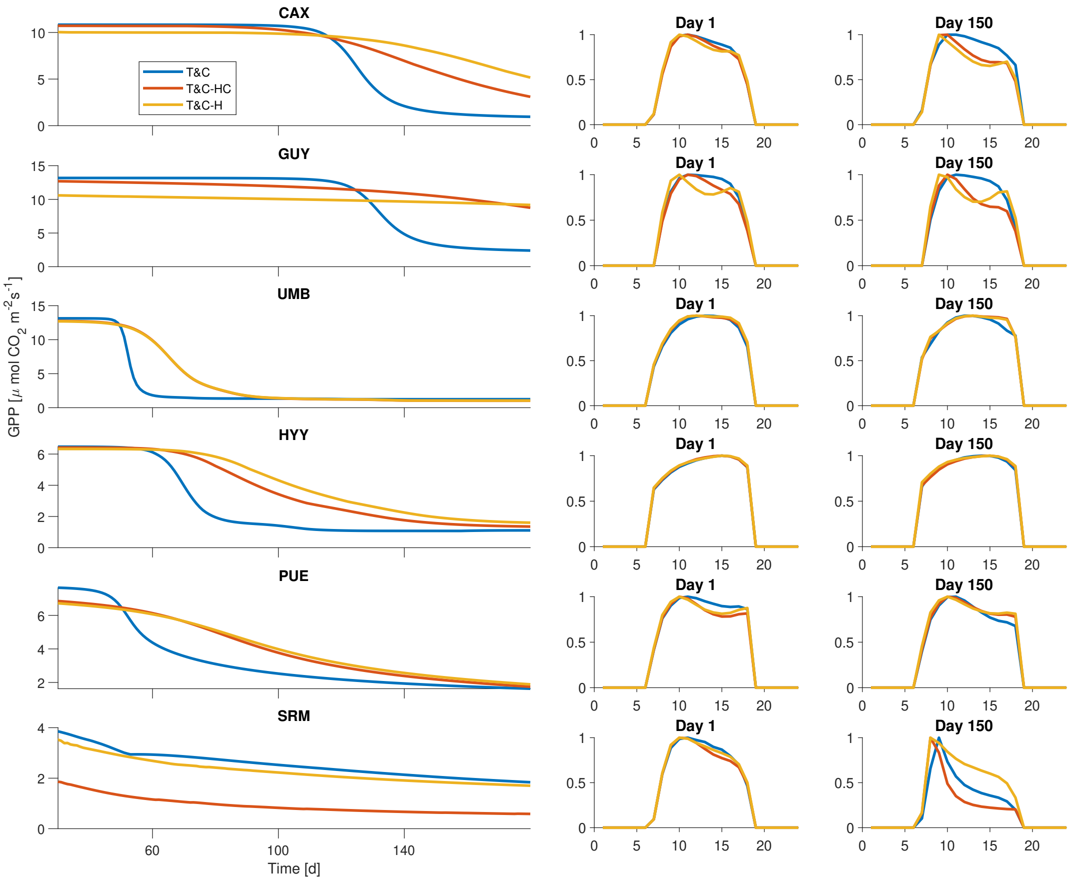

Figure S4: Same as Figure 2 in the main paper, but for all sites under E1b

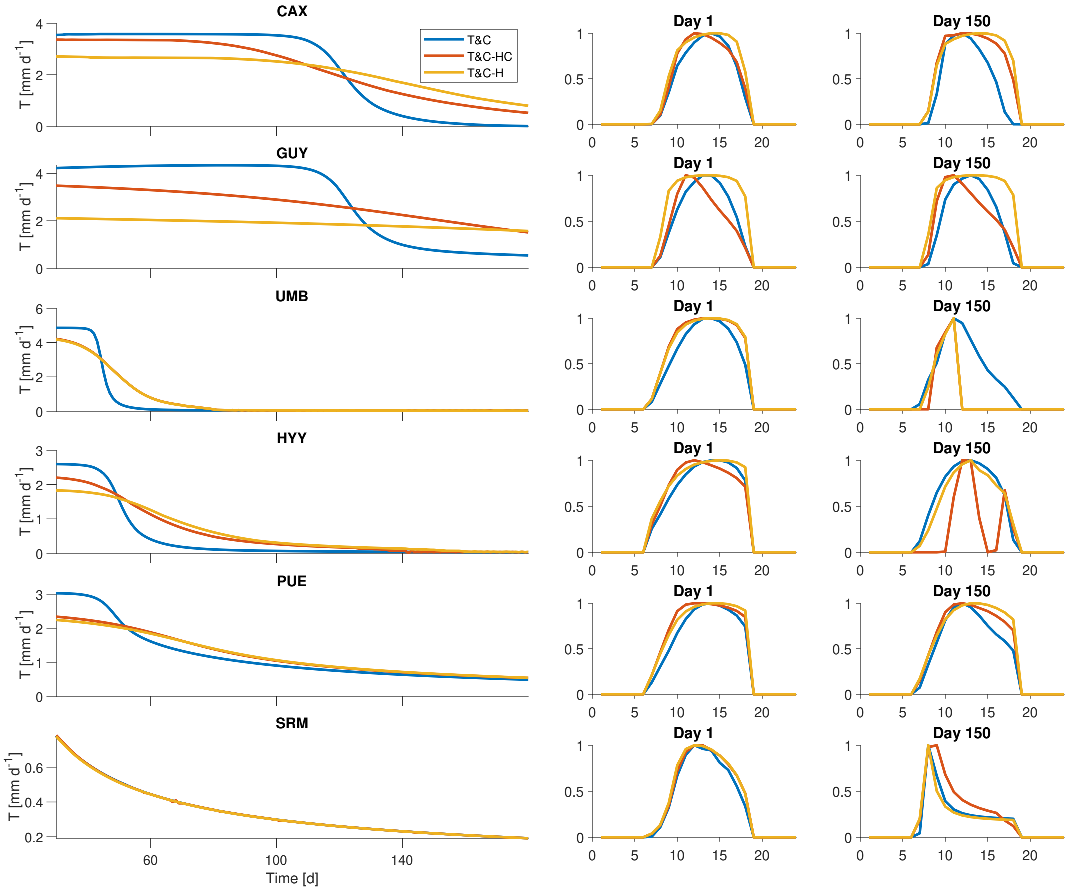

Figure S5: Same as Figure 2 in the main paper, but for plant transpiration for all sites under E1a

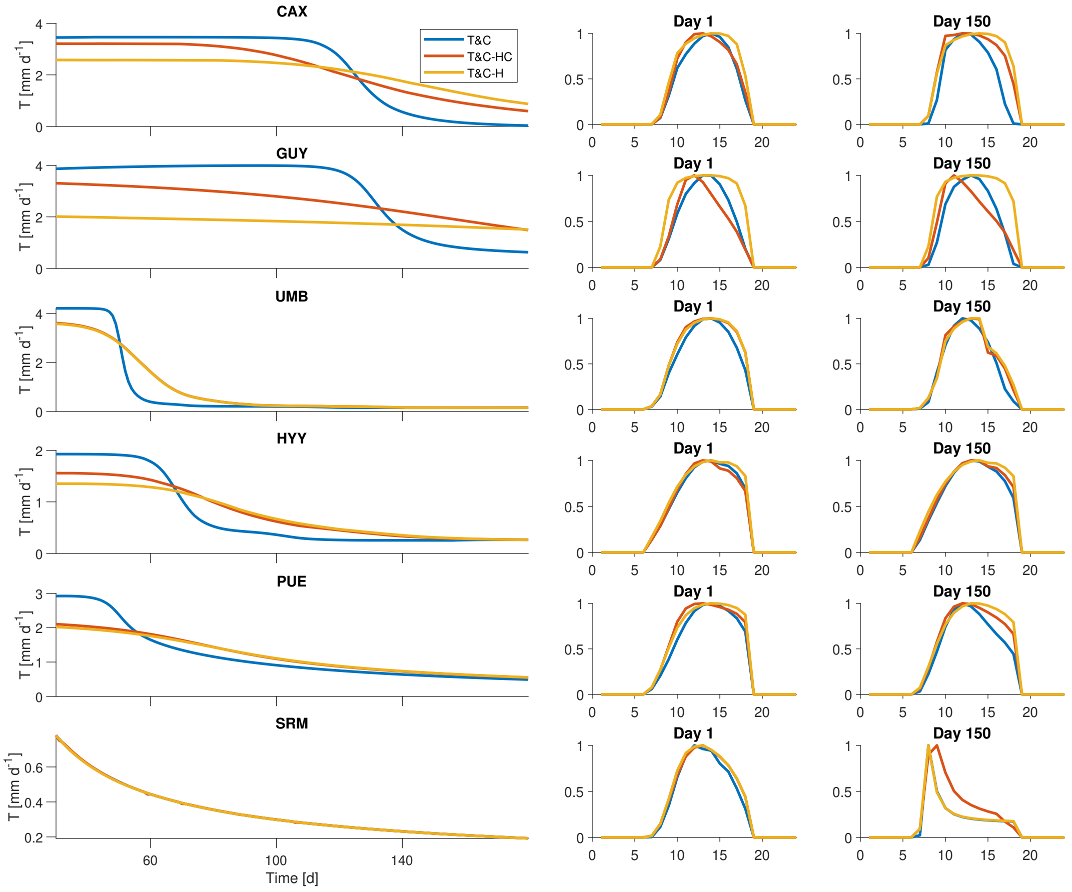

Figure S6: Same as Figure 2 in the main paper, but for plant transpiration for all sites under E1b

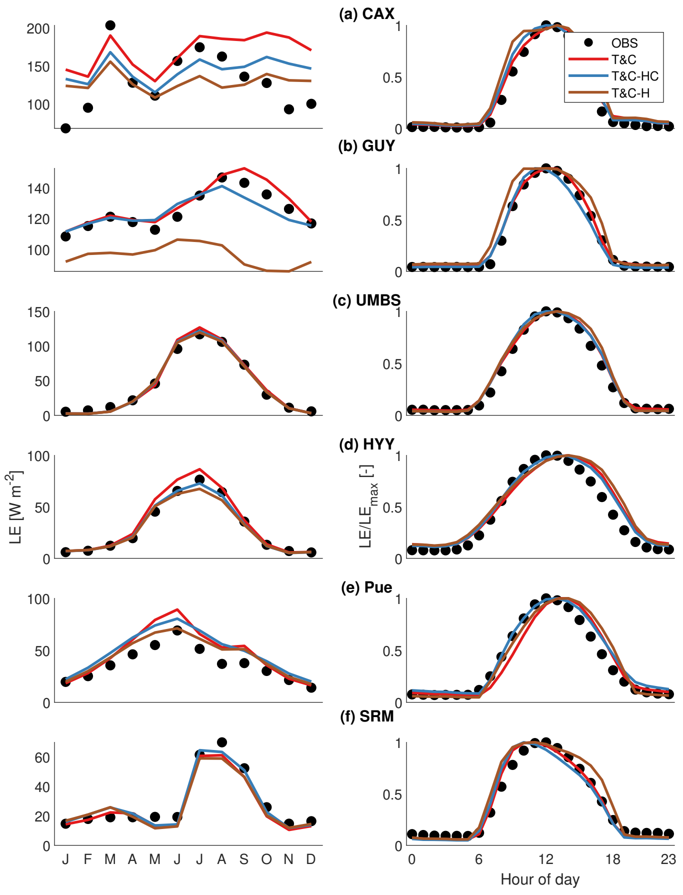

Figure S7: Same as Figure 5 in the main paper, but for latent heat fluxes

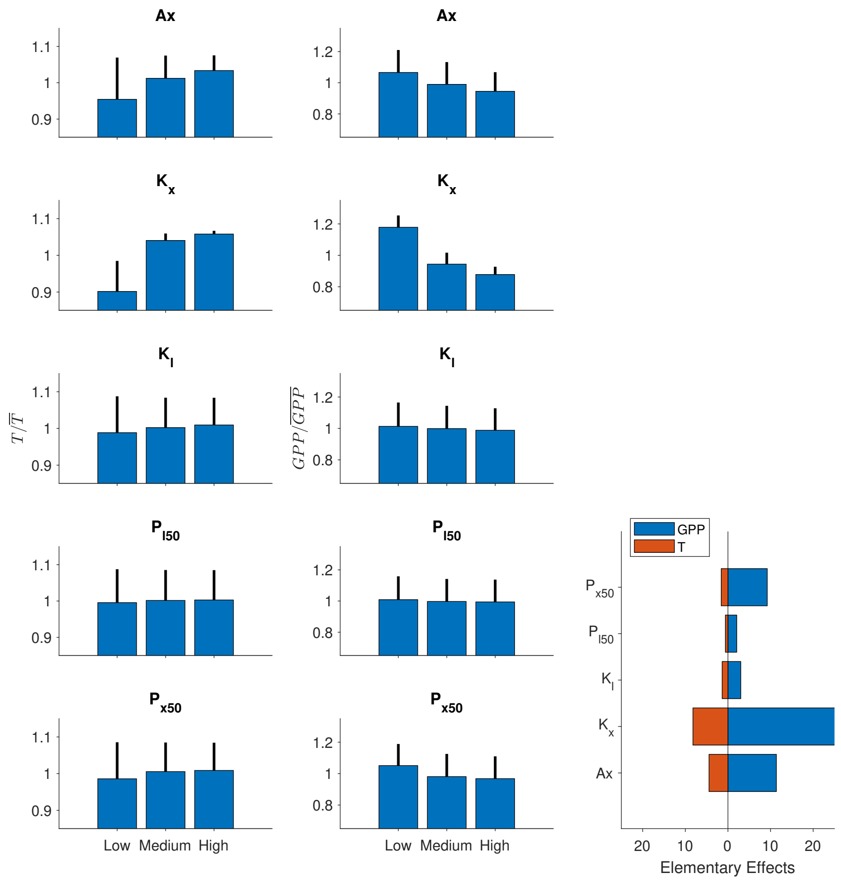

Figure S8: Results of the model parameter sensitivity analysis performed using the T&C-HC model for the E1a experiment for the site FR-Pue. The sensitivity analysis run the E1a experiment but with the following parameter combinations: $A_x = [4, 8, 12] cm^2 stem/m^2$, $Kx_{max} = [10^5, 5 * 10^5, 10^6] mmol H_2 0 m^{-1} s^{-1} MPa^{-1}$, $Kl_{max} = [2, 5, 10] mmol H_2 0 m^{-2} s^{-1} MPa^{-1}$, $\psi_{l50} = [-1, -2, -3] MPa$, $\psi_{x50} = [-1, -2, -3] MPa$. All parameter combinations were modelled. The errorbars show the ratio between the average transpiration (left) and GPP (middle) for each parameter choice and the overall average transpiration for all parameter combinations. On the right an estimate of the parameter sensitivity expressed using the Elementary Effects method (Pappas et al., 2013)

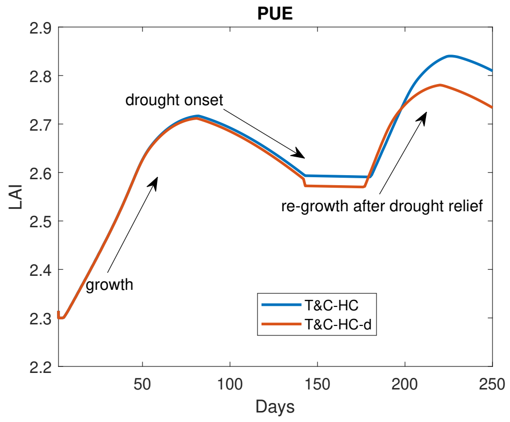

Figure S9: Simulated leaf area index (LAI) for the FR-Pue site using the T&C-HC and T&C-HC-d for the E2a experiment.

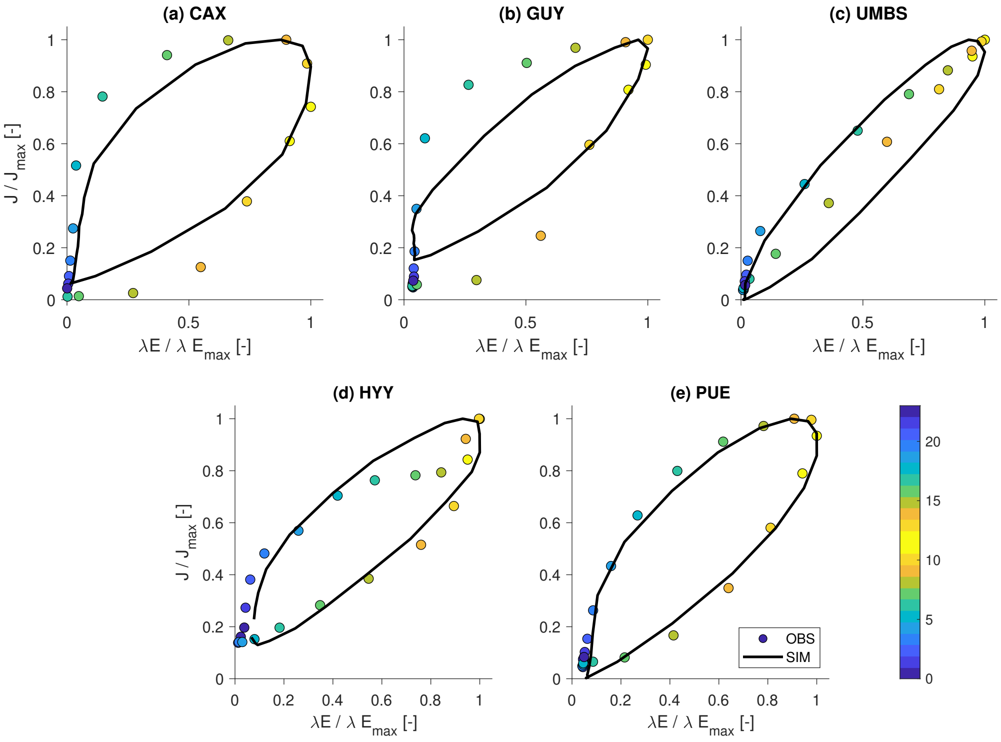

Figure S10: Hysteretical loop between observed (dots) and simulated with the T&C-HC model (lines) between standardized latent heat fluxes and sapflux during the day. J refers to average sapflow for each hour, J_{max} to the highest value of J. λE refers to the average latent heat flux for each hour, and λE_{max} to the highest value of λE .

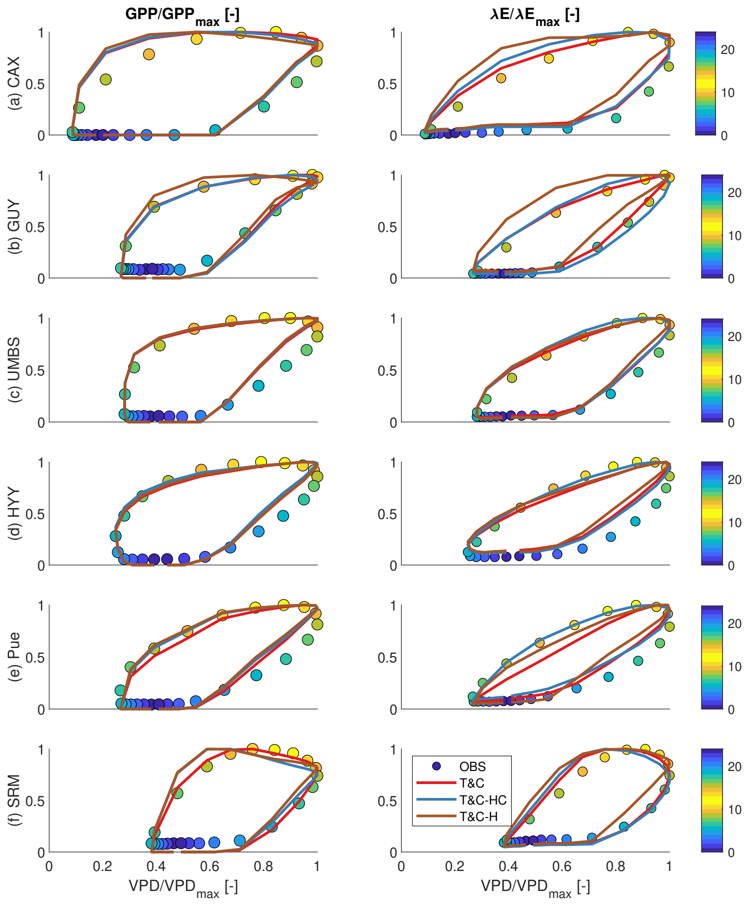

Figure S11: Similar to Figure S10. Hysteretical loop between the standardized Gross Primary Productivity (left) and latent heat flux (right), and vapor pressure deficit. GPP, λE and VPD refer to average hourly values of gross primary productivity, latent heat flux and vapor pressure deficit. GPP$_{m}ax$, $\lambda E_{m}ax$ and VPD$_{m}ax$ refet to the maximum values of GPP, λE and VPD. Dots refer to observations from flux tower data, and lines to model simulations.

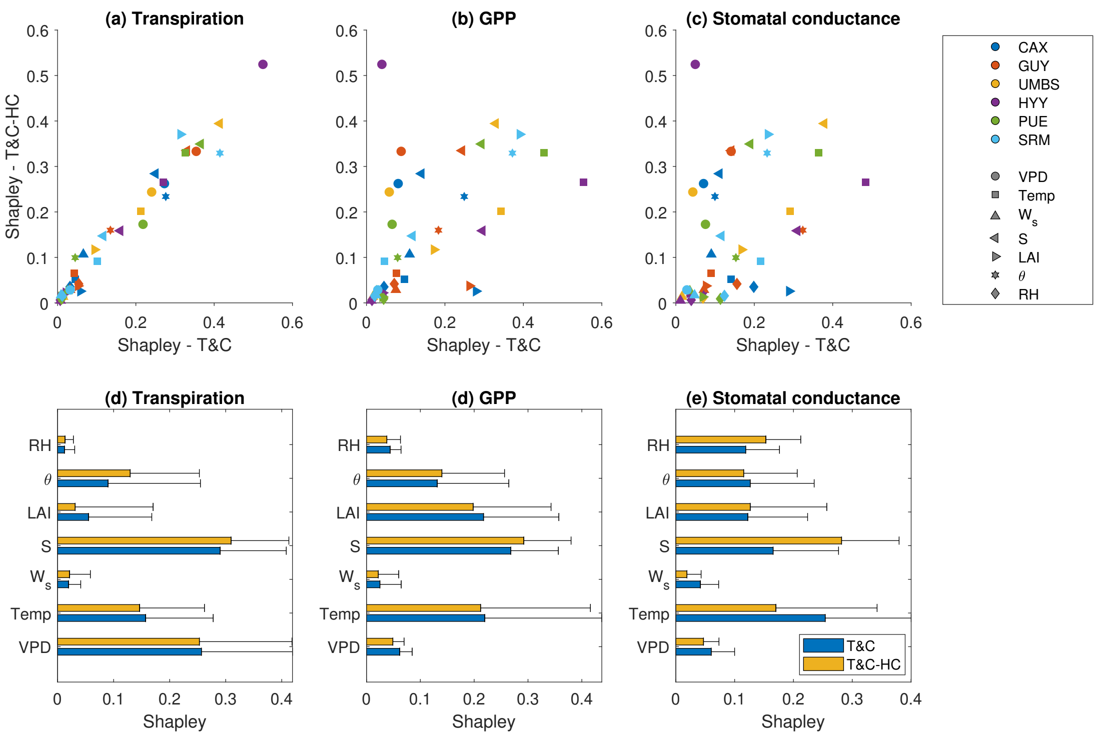

Figure S12: Same as Figure 8 in the main paper, but for the Shapley values instead.

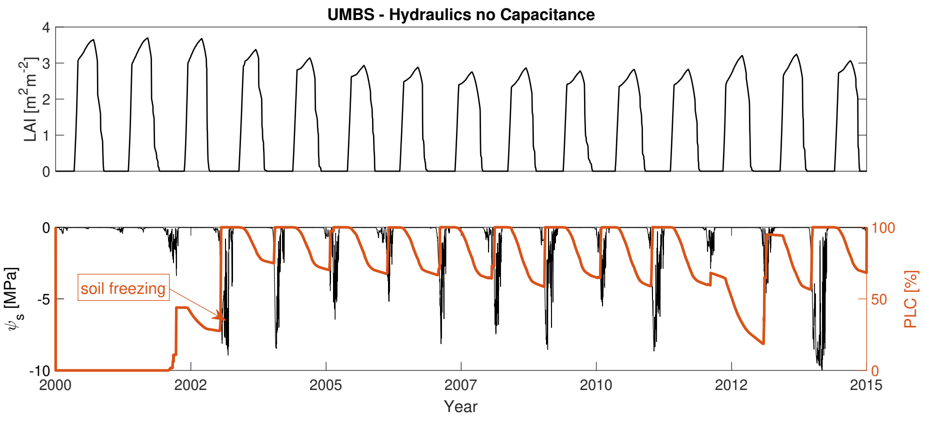

Figure S13: (a) Simulated leaf area index (LAI) for the US-UMB site using the T&C-HC-d model. (b) Simulated soil water potential (black lines) and percentage loss of xylem conductivity (orange line) for the US-UMB site using the T&C-HC-d model

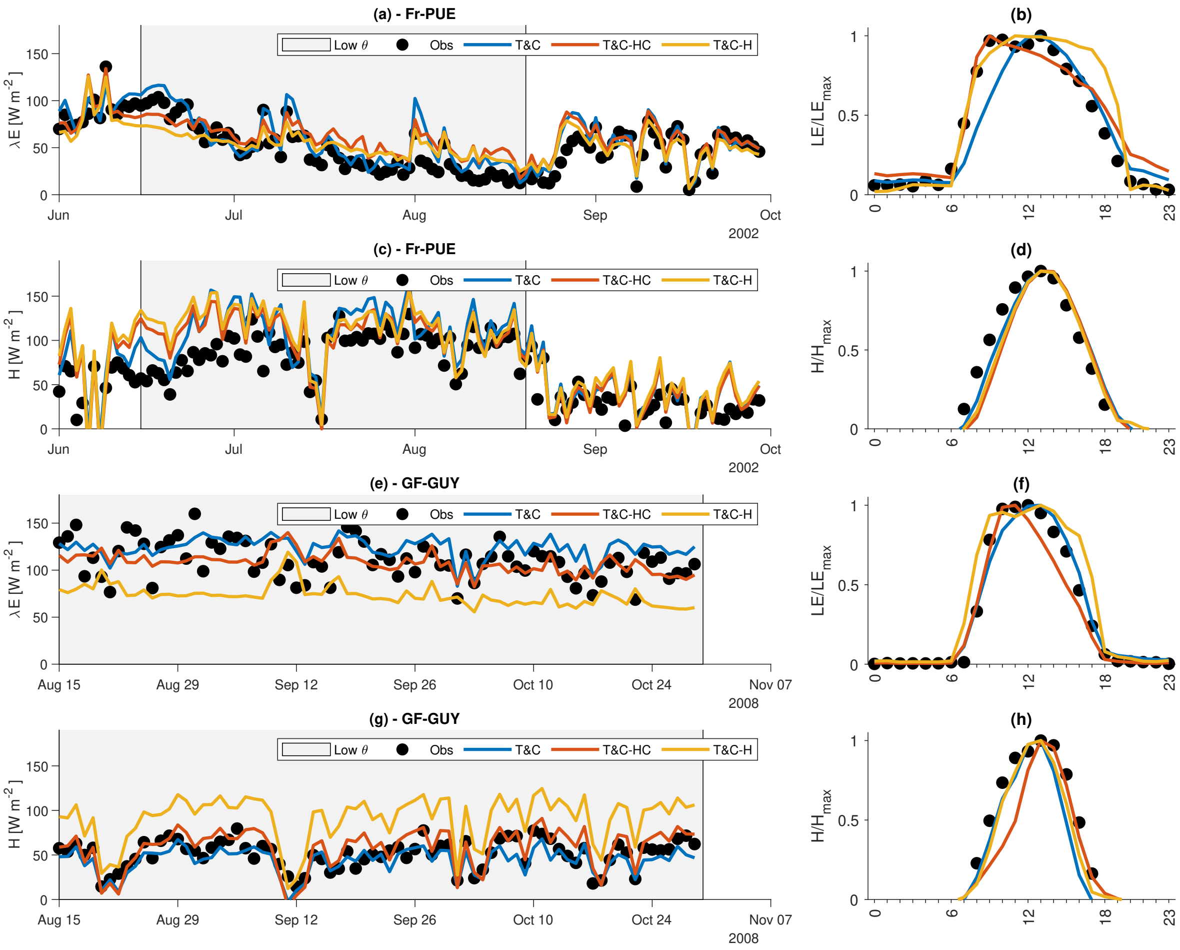

Figure S14: Same as Figure 5 but for latent and sensible heat fluxes.

		Br-CAX	GF-GUY	US-UMB	FI-HYY	FR-PUE	US-SRM	
							PFT1	PFT2
Common	Photosynthetic pathway	C3	C3	C3	C3	C3	C3	C3
	$V_{cmax}[\mu molm^{-2}s^{-1}]$	52	54	65	40	44	60	59
	$J_{max}[\mu molm^{-2}s^{-1}]$	114.4	118.8	156	76	92.4	120	118
	Root depth [mm]	3000	5000	2000	700	300	1000	600
	$\psi_{s50}[MPa]$	-1.7	-1.7	-1.5	-2.5	-1.3	-3.5	-2
	$\psi_{s10}[MPa]$	-0.3	-0.3	-0.2	-0.4	-0.3	-0.3	-0.4
T&C	a1 [-]	6	6	6	8	6	8	4
	D0 [Pa]	1000	1000	1000	1000	1000	1400	1000
T&C-HC	a1 [-]	4	4	4	5	3	5	2
and T&C-H	$\psi_{x50}[MPa]$	-2	-3	-3.5	-3.5	-3.5	-4.5	-2.5
	$\psi_{x10}[MPa]$	-0.5	-0.6	-0.6	-0.6	-0.5	-0.7	-0.5
	$\psi_{l50}[MPa]$	-0.8	-1.9	-1.5	-2.3	-1.5	-3.5	-1.5
	$\psi_{l10}[MPa]$	-0.2	-0.3	-0.3	-0.3	-0.3	-0.4	-0.3
	$k_x^{max}[mmolm^{-1}s^{-1}MPa^{-1}]$	200000	100000	50000	27000	35000	20000	50000
	$k_l^{max}[mmolm^{-2}s^{-1}MPa^{-1}]$	10	10	5	5	5	3	5
	c_x [MPa]	-5	-6	-8	-7	-8	-10	-8
	c_l [MPa]	-2	-3	-4	-5	-6	-7	-5
T&C-HC and T&C-H	Parameters informed from	(Giles et al., 2022)	(Santiago et al., 2018)	(Li et al., 2021)	(Duursma et al., 2008)	(Limousin et al., 2010)	(Hultine et al., 2006)	

* ψ_{10} and ψ_{50} refer to water potentials at 10% and 50% loss of conductivity, or it the case of soil the water potentials causing $_s = 0.9$ and $_s = 0.5$

Underscores $_s$, $_x$, $_l$ in the ψ_{50} and ψ_{10} parameters correspond to soil, xylem and leaf accordingly

** The parameters p and q in equations 5,6,9 in the main manuscript were computed by ψ_{10} and ψ_{50} as:

$p = 1.8839/(\ln(\psi_{50}) - \ln(\psi_{10}))$

$q = 0.6931^{1/p}/(-\psi_{50})$

References

Duursma, R. A., P. Kolari, M. Perämäki, E. Nikinmaa, P. Hari, S. Delzon, D. Loustau, H. Ilvesniemi, J. Pumpanen, and A. Mäkelä (2008). Predicting the decline in daily maximum transpiration rate of two pine stands during drought based on constant minimum leaf water potential and plant hydraulic conductance. *Tree physiology 28*(2), 265–276.

Giles, A., L. Rowland, P. Bittencourt, D. Bartholomew, I. Coughlin, P. Costa, T. Domingues, R. Miatto, F. Barros, L. Ferreira, et al. (2022). Small understorey trees have greater capacity than canopy trees to adjust hydraulic traits following prolonged experimental drought in a tropical forest. *Tree physiology 42*(3), 537–556.

Hultine, K., D. Koepke, W. Pockman, A. Fravolini, J. Sperry, and D. Williams (2006). Influence of soil texture on hydraulic properties and water relations of a dominant warm-desert phreatophyte. *Tree Physiology 26*(3), 313–323.

Li, L., Z.-L. Yang, A. M. Matheny, H. Zheng, S. C. Swenson, D. M. Lawrence, M. Barlage, B. Yan, N. G. McDowell, and L. R. Leung (2021). Representation of plant hydraulics in the noah-mp land surface model: Model development and multiscale evaluation. *Journal of Advances in Modeling Earth Systems 13*(4), e2020MS002214.

Limousin, J.-M., D. Longepierre, R. Huc, and S. Rambal (2010). Change in hydraulic traits of mediterranean quercus ilex subjected to long-term throughfall exclusion. *Tree Physiology 30*(8), 1026–1036.

Pappas, C., S. Fatichi, S. Leuzinger, A. Wolf, and P. Burlando (2013). Sensitivity analysis of a process-based ecosystem model: Pinpointing parameterization and structural issues. *Journal of Geophysical Research: Biogeosciences 118*(2), 505–528.

Santiago, L. S., M. E. De Guzman, C. Baraloto, J. E. Vogenberg, M. Brodie, B. Hérault, C. Fortunel, and D. Bonal (2018). Coordination and trade-offs among hydraulic safety, efficiency and drought avoidance traits in amazonian rainforest canopy tree species. *New Phytologist 218*(3), 1015–1024.
